# Disease and brain region specific immune response profiles in neurodegenerative diseases with pure and mixed protein pathologies

**DOI:** 10.1186/s40478-024-01770-7

**Published:** 2024-04-05

**Authors:** Tim Bathe, Gabriela P. Hery, Jonathan A. B. Villareal, Jennifer L. Phillips, Eric M. Cohen, Rohan V. Sharma, Wangchen Tsering, Stefan Prokop

**Affiliations:** 1 Department of Pathology, Immunology & Laboratory Medicine, University of Florida, Gainesville, FL 32610 USA; 2 Center for Translational Research in Neurodegenerative Disease, University of Florida, Gainesville, FL 32610 USA; 3 Department of Small Animal Clinical Sciences, College of Veterinary Medicine, University of Florida, Gainesville, FL 32608 USA; 4 Department of Neuroscience, University of Florida, Gainesville, FL 32610 USA; 5 J. Crayton Pruitt Family Department of Biomedical Engineering, Herbert Wertheim College of Engineering, University of Florida, Gainesville, FL 32611 USA; 6 Fixel Institute for Neurological Diseases, University of Florida, Gainesville, FL 32608 USA; 7 McKnight Brain Institute, University of Florida, Gainesville, FL 32610 USA

**Keywords:** Microglia, Alzheimer’s disease, Protein pathology, Amyloid beta plaques, Digital spatial profiling, Deep immunophenotyping, Mixed pathologies

## Abstract

The disease-specific accumulation of pathological proteins has long been the major focus of research in neurodegenerative diseases (ND), including Alzheimer’s disease (AD) and related dementias (RD), but the recent identification of a multitude of genetic risk factors for ND in immune-associated genes highlights the importance of immune processes in disease pathogenesis and progression. Studies in animal models have characterized the local immune response to disease-specific proteins in AD and ADRD, but due to the complexity of disease processes and the co-existence of multiple protein pathologies in human donor brains, the precise role of immune processes in ND is far from understood. To better characterize the interplay between different extracellular and intracellular protein pathologies and the brain’s intrinsic immune system in ND, we set out to comprehensively profile the local immune response in postmortem brain samples of individuals with “pure” beta-Amyloid and tau pathology (AD), “pure” α-Synuclein pathology in Lewy body diseases (LBD), as well as cases with Alzheimer’s disease neuropathological changes (ADNC) and Lewy body pathology (MIX). Combining immunohistochemical profiling of microglia and digital image analysis, along with deep immunophenotyping using gene expression profiling on the NanoString nCounter® platform and digital spatial profiling on the NanoString GeoMx® platform we identified a robust immune activation signature in AD brain samples. This signature is maintained in persons with mixed pathologies, irrespective of co-existence of AD pathology and Lewy body (LB) pathology, while LBD brain samples with “pure” LB pathology exhibit an attenuated and distinct immune signature. Our studies highlight disease- and brain region-specific immune response profiles to intracellular and extracellular protein pathologies and further underscore the complexity of neuroimmune interactions in ND.

**Supplementary Information:**

The online version contains supplementary material available at 10.1186/s40478-024-01770-7.

## Introduction

Alzheimer's disease (AD) is the most common form of dementia worldwide [2, 50]. It affects over 55 million people and impacts not only patients, but also their families and caregivers [91]. Pathologically, the hallmarks of AD are extracellular deposits of amyloid beta (Aβ) in form of plaques, intracellular aggregates of tau in form of neurofibrillary tangles (NFT) and neuronal loss. More recently a prominent response of the local immune system including astrogliosis and microgliosis has been recognized as a key component of AD pathophysiology [7, 13, 27, 29, 46, 48, 68, 82, 86]. Activation of microglia, particularly around Aβ deposits, is observed in AD and has been studied extensively in animal models [63, 64]. These studies have established gene expression signatures of activated microglia in response to Aβ deposition [21, 23, 35, 44, 72, 89] and have demonstrated the impact of genetic AD risk variants in microglia-associated genes on microglia activation [38, 65, 92]. While walling off and phagocytosing of Aβ plaques [21, 29, 59] by microglia is considered beneficial, activation-associated cytokine secretion and overactivation-associated phagocytosis of synapses have been linked to exacerbating disease progression [1, 3]. Thus, it is still a matter of hot debate whether stimulating microglia or inhibiting microglia is the more promising therapeutic approach to combat Aβ deposits.

The immune response to prominent intracellular protein pathologies, such as tau or α-Synuclein (α-Syn) pathology has also been studied and has revealed a more subtle, yet prominent activation of microglia in animal models of tauopathy or α-Synucleinopathy [6, 14, 52, 60]. Translating these groundbreaking studies to the human disease condition has been successful, especially with respect to the activation signatures reported in response to Aβ [51, 88, 93]. Cross-species comparisons however have also documented key differences between model systems and the human disease condition, which are important to consider when moving towards therapeutic interventions. One key difference is prominent age-related changes observed in humans, which can only be modeled in a limited way in animal models [15, 55, 69, 74]. Certain age-related phenotypes in microglia are only observed in humans, such as microglia dystrophy, characterized by beaded microglia processes, changes in nuclear morphology, and an arrested functional state [42, 45, 76–78]. Furthermore, ND rarely present with “pure” pathology, restricted to one or two protein pathologies. In fact, the majority of patients who come to autopsy exhibit multiple protein pathologies at the time of death [66, 67]. Previous studies have investigated the heterogeneity of microglia in different brain regions. In these studies, the assessment of microglial activation phenotypes was limited to single disease backgrounds in the mouse or human brain [8, 10, 20, 56]. However, the interaction of occurrence of co-pathologies and brain region-specific microglial activation has not been investigated. It is therefore of utmost importance to study the innate immune response in the complex environment of the diseased human brain in order to successfully translate studies in animal models to therapeutic interventions in humans.

We have previously studied the innate immune response in cases with pure (only Aβ and tau) AD pathology, showing that microglia activation follows the progression of Aβ plaques and NFT, while Aβ pathology or NFT pathology alone were not sufficient to drive a strong microglia response [65]. Expanding this study to the complex landscape of ND, we set out to compare and contrast the microglia response in pure AD cases (AD) with the response in cases with intracellular Lewy body disease pathology (LBD) and cases with mixed AD and LBD pathology (MIX).

## Methods

### Human brain tissue

Brain specimens from 93 brain donors were provided by the University of Florida Neuromedicine Human Brain and Tissue Bank (UF HBTB). Neuropathological findings were evaluated according to current guidelines for postmortem diagnosis of AD, LBD, LATE, and other associated pathologies by a board-certified neuropathologist. To categorize the severity of the disease of the subjects used in this study, cases were scored according to their Alzheimer’s disease neuropathological changes (ADNC). The ADNC assessment used three different measures to evaluate the presence and severity of AD: the Thal phase of Aβ deposition, the Braak stages for tau neurofibrillary pathology, and the CERAD score for neuritic plaque density [9, 54, 83]. The ADNC was categorized into three levels: low, intermediate, and high, based on the combined ABC score [25]. These scales measure Aβ plaques (A) using the Thal phases method, NFT stage (B) using the Braak method, and neuritic plaque score (C) using the CERAD method. The combination of A, B, and C scores can be classified as "Not," "Low," "Intermediate," or "High" AD neuropathologic change. The presence of "Intermediate" or "High" AD neuropathologic change is typically sufficient to explain the dementia associated with AD [25]. Finally, primary age-related tauopathy (PART) was classified as present in participants with definite PART, as defined by Braak Stage I-IV, and no neuritic plaques [12]. According to the pathological findings, study subjects were divided into four groups: cases without significant pathological changes (n = 23) were classified as “Control”. AD cases, that were after the Braak stages classified as cases with high ADNC, such as Aβ plaques and NFT, without other significant co-pathologies were grouped into “AD” (n = 29). Cases with a predominant Lewy body pathology (n = 19) were called “LBD”. To evaluate the innate immune response to different protein pathologies combined in one disease classification, cases with both Lewy body pathology and high ADNC (n = 22) were grouped as “MIX”. This study focused on cases with a high disease burden according to the ADNC scoring. Therefore, AD and MIX cases with low or intermediate burden were not included in diease groups. To ensure the best possible comparison, care was taken to ensure that the individual cases were as similar in age as possible. This resulted in the following medians and standard deviations for the study groups: Control 75 (± 11), MIX 78 (± 9), AD 78 (± 9), and LBD 80 (± 8). In addition, the APOE genotypes of the individuals were collected. Because of its role as a major risk factor for AD, the APOE genotype was also examined. Therefore, cases with at least one APOE E4 allele were distributed among the groups as follows: Control 5%, MIX 68%, AD 46%, and LBD 16%. The selection of cases was limited by the availability of brains from the brain bank. For each of these cases, tissue from disease-relevant regions like frontal cortex (FC), hippocampus (HPC), occipital cortex (OCC) and midbrain (MB) were evaluated. Age, gender, disease group, and APOE genotype are shown in Table 1.

**Table 1:** Study cohort data and neuropathological scoring. Cases were selected by pathology and grouped into four cohorts: Control; MIX; AD; LBD. Neuropathological scores were evaluated by a board-certified neuropathologist

Case #	Study group	Pathological diagnosis	Thal	Braak	Cerad	LB Score (*McKeith*)	APOE	Gender	Age	PMI	Exp
1	Control	PART, definite, Braak II	0	II	None	None	3/3	m	85	NA	1
2	Control	Atherosclerosis (moderate)	0	0	None	None	3/3	m	71	NA	1
3	Control	Atherosclerosis (moderate)	0	0	None	None	3/3	m	88	5	1
4	Control	PART, definite, Braak I	0	I	None	None	3/3	m	87	NA	1
5	Control	PART, definite, Braak I	0	I	None	None	3/3	m	77	NA	1
6	Control	Acute microinfarcts	0	0	None	None	3/3	m	72	4	1
7	Control	PART, definite, Braak I	0	I	None	None	2/3	f	73	NA	1
8	Control	PART, definite, Braak II	0	II	None	None	N/A	f	82	NA	1
9	Control	PART, definite, Braak II	0	II	None	None	3/3	f	90	NA	1
10	Control	PART, definite, Braak I	0	I	None	None	3/3	f	78	5	1
11	Control	PART, definite, Braak I	0	I	None	None	2/3	m	74	5	1
12	Control	PART, definite, Braak II	0	II	None	None	3/3	m	77	6	1
13	Control	PART, definite, Braak I	0	I	None	None	2/3	m	51	NA	1
14	Control	PART, definite, Braak I	0	I	None	None	2/3	m	52	NA	1
15	Control	Atherosclerosis (moderate)	1	I	None	None	3/3	m	84	NA	1,2
16	Control	PART, Braak I	0	I	None	None	3/3	f	76	10	1,2
17	Control	PART, Braak I	0	I	None	None	3/3	m	71	6	1,2,3
18	Control	AD low	1	II	None	None	3/3	m	90	14	1,2
19	Control	AD low	1	II	None	None	3/3	m	88	4	1,2
20	Control	PART, definite, Braak II	0	II	None	None	3/3	f	72	4	1,2,3
21	Control	CVD	1	II	None	None	3/3	f	90	56	1,3
22	Control	PART, definite, Braak II	0	II	None	None	3/3	m	77	12	1,2
23	Control	Control	0	0	None	None	3/4	f	55	12	1,2,3
24	MIX	AD high	5	VI	Frequent	Diffuse neocortical	4/4	f	78	13	1
25	MIX	AD high	5	VI	Frequent	Diffuse neocortical	3/4	m	78	22	1
26	MIX	AD high	5	VI	Frequent	Diffuse neocortical	4/4	m	90+	14.25	1
27	MIX	AD high	4	V	Frequent	Diffuse neocortical	3/4	f	76	20	1,3
28	MIX	AD high	5	VI	Frequent	Diffuse neocortical	3/3	m	83	69	1
29	MIX	AD high	5	VI	Frequent	Diffuse neocortical	3/3	m	62	5	1,3
30	MIX	AD high	5	VI	Frequent	Limbic-transitional	4/4	f	76	3	1
31	MIX	AD high	5	VI	Frequent	Limbic-transitional	3/3	f	81	7	1
32	MIX	AD high	5	VI	Frequent	Limbic-Transitional	3/4	m	64	6	1
33	MIX	AD high	5	V	Frequent	Limbic-transitional	3/3	f	78	12	1
34	MIX	AD high	5	V	Frequent	Diffuse neocortical	3/3	f	63	12	1
35	MIX	AD high	5	V	Frequent	Diffuse neocortical	2/3	f	90+	9	1
36	MIX	AD high	5	V	Frequent	Diffuse neocortical	4/4	f	90+	18	1
37	MIX	AD high	5	VI	Frequent	Diffuse neocortical	4/4	f	75	NA	1,3
38	MIX	AD high	5	V	Frequent	Limbic-Transitional	4/4	f	85	NA	1
39	MIX	AD high	5	V	Frequent	Diffuse neocortical	3/4	m	74	7	1
40	MIX	AD high	5	VI	Frequent	Diffuse neocortical	3/3	m	87	9	1,2,3
41	MIX	AD high	4	V	Frequent	Diffuse neocortical	3/4	m	77	17	1,2,3
42	MIX	AD high	5	V	Frequent	Diffuse neocortical	3/4	f	83	6	1,2,3
43	MIX	AD high	5	V	Frequent	Diffuse neocortical	3/4	m	80	21	1,2,3
44	MIX	AD high	5	V	Frequent	Diffuse neocortical	2/4	f	74	8	1,2
45	MIX	AD high	5	V	Frequent	Diffuse neocortical	3/4	f	68	6	1,2,3
46	AD	AD high	5	VI	Frequent	None	3/4	m	81	4	1
47	AD	AD high	5	VI	Frequent	None	3/4	m	63	2	1
48	AD	AD high	5	VI	Frequent	None	3/3	m	66	5	1,3
49	AD	AD high	4	V	Frequent	None	3/4	m	77	5	1
50	AD	AD high	5	V	Frequent	None	2/4	f	90+	10	1
51	AD	AD high	5	V	Frequent	None	3/3	m	90+	5	1
52	AD	AD high	5	VI	Frequent	None	3/3	m	83	7	1
53	AD	AD high	5	VI	Frequent	None	3/4	m	59	7	1,3
54	AD	AD high	5	V	Frequent	None	3/3	f	64	12	1
55	AD	AD high	5	VI	Frequent	None	4/4	m	74	36	1
56	AD	AD high	5	V	Frequent	None	3/4	f	86	14	1
57	AD	AD high	5	VI	Frequent	None	3/3	m	60	20	1
58	AD	AD high	5	VI	Frequent	None	4/4	m	70	8	1,3
59	AD	AD high	5	V	Frequent	None	2/3	m	74	2	1
60	AD	AD high	4	VI	Frequent	None	4/4	m	75	NA	1
61	AD	AD high	5	VI	Frequent	None	N/A	f	75	NA	1
62	AD	AD high	5	VI	Frequent	None	3/3	f	86	6	1
63	AD	AD high	5	VI	Frequent	None	3/3	f	84	NA	1,3
64	AD	AD high	5	V	Frequent	None	N/A	f	82	NA	1
65	AD	AD high	5	V	Frequent	None	3/3	f	90	NA	1
66	AD	AD high	5	V	Frequent	None	3/3	m	87	NA	1
67	AD	AD high	5	V	Frequent	None	3/3	m	73	NA	1
68	AD	AD high	4	V	Frequent	None	3/4	m	84	16	1,2
69	AD	AD high	4	V	Frequent	None	3/4	f	85	18	1
70	AD	AD high	5	V	Frequent	None	3/4	f	72	12	1,2
71	AD	AD high	5	V	Frequent	None	3/3	f	78	5	1,2
72	AD	AD high	4	V	Frequent	None	3/3	f	78	20	1
73	AD	AD high	5	V	Frequent	None	3/4	m	78	20	1,2
74	AD	AD high	5	VI	Frequent	None	3/3	m	83	18	1,2
75	LBD	LBD	3	II	None	Limbic-transitional	3/3	m	83	18	1
76	LBD	LBD	3	II	Sparse	Diffuse neocortical	3/4	m	68	8	1,3
77	LBD	LBD	3	I	None	Diffuse neocortical	2/3	m	69	3	1
78	LBD	LBD	2	I	None	Brainstem predominant	3/3	m	77	NA	1
79	LBD	LBD	0	I	None	Diffuse neocortical	2/3	m	80	3	1,3
80	LBD	LBD	2	II	None	Limbic-transitional	3/3	f	86	11	1,3
81	LBD	LBD	3	I	None	Brainstem predominant	3/3	m	57	17	1,3
82	LBD	LBD	2	I	None	Brainstem predominant	3/3	m	84	13	1,3
83	LBD	LBD	3	II	None	Diffuse neocortical	3/3	f	67	8	1
84	LBD	LBD	3	II	Sparse	Brainstem predominant	2/3	m	90+	9	1,3
85	LBD	LBD	1	II	None	Limbic-transitional	3/4	m	75	12	1
86	LBD	LBD	3	II	None	Diffuse neocortical	3/4	m	80	7	1
87	LBD	LBD	3	II	None	Diffuse neocortical	3/3	m	83	NA	1
88	LBD	LBD	3	II	None	Diffuse neocortical	3/3	m	76	11	1,2
89	LBD	LBD	3	II	Moderate	Diffuse neocortical	3/3	m	80	4	1,2,3
90	LBD	LBD	3	II	Sparse	Diffuse neocortical	3/3	m	79	< 15	1,2,3
91	LBD	LBD	0	II	None	Diffuse neocortical	3/3	m	85	12	1,2,3
92	LBD	LBD	1	II	None	Diffuse Neocortical	3/3	m	62	10	1,2,3
93	LBD	LBD	1	II	Sparse	Diffuse neocortical	3/3	m	81	3	1,2

*Thal* Thal phases; *Braak* Braak stages; *CERAD* Neuritic plaque score; LB Score (McKeith) Lewy body scoring after *Ian G. McKeith*; *APOE* APOE genotype; *CVD* Cerebral vascular dementia. Exp:1– IHC, 2 – nCounter, 3 – GeoMx DSP

### Immunohistochemistry

5 μm (pathological staining) and 10 μm (microglial staining) thick tissue sections of formalin-fixed, paraffin-embedded (FFPE) brain tissue specimens were rehydrated in Xylene and descending alcohol series. Heat-induced epitope retrieval (HIER) was performed in a pressure cooker (Tintoretriever, Bio SB) for 15 min at high pressure in a 0.05% Tween-20 solution. Endogenous peroxidase was quenched by incubation of sections in 30% hydrogen peroxide/10% Triton-X-100 diluted in pH 7.6 sterile phosphate buffered saline (PBS) (Invitrogen) for 20 min, following multiple washes in tap water and subsequently, 0.1 M Tris, pH 7.6. Non-specific antibody binding was minimized with sections incubated in 2% FBS/0.1 M Tris, pH 7.6. Primary antibodies (Table 4) were diluted in 2% FBS/0.1 M Tris, pH 7.6. Sections were incubated with primary antibody over night at 4 °C, washed one time in 0.1 M Tris, pH 7.6, followed by 2% FBS/0.1 M Tris, pH 7.6 for 5 min, and incubated in goat anti-rabbit IgG HRP conjugated secondary antibody (Millipore Sigma) for 1 h. Additionally, their were washed one time in 0.1 M Tris, pH 7.6, followed by 2% FBS/0.1 M Tris, pH 7.6 for 5 min, and incubated in VectaStain ABC Peroxidase HRP Kit (diluted in 2% FBS/0.1 M Tris, pH 7.6 at 1:1000) for 1 h. After a final wash in 0.1 M Tris, pH 7.6 for 5 min, immunocomplexes were visualized using the Vector Laboratories ImmPACT DAB Peroxidase (HRP) 3,3′- diaminobenzidine. Tissue sections were counterstained with hematoxylin (Sigma Aldrich, St. Louis, MO) for 2 min, dehydrated in ascending alcohol series and Xylene and cover slipped using Cytoseal 60 mounting medium (Thermo Scientific).

### Pathology and microglia quantification

For analysis of stains, slides were scanned on an Aperio AT2 scanner (Leica Biosystems) at 20x (protein pathology) and 40x (microglia quantification) magnification. Digital slides were analyzed using the QuPath platform (version 0.3.2, https://QuPath.github.io/) [5], on a Dell PC (Intel® Xeon® W-1270 CPU @ 3.40 GHz/ 64 GB RAM/ 1 TB SSD Hard Drive) running Windows 10. Regions of interest (ROIs) were annotated for regional analysis. The annotation and quantification of the analyzed regions focused on the gray matter areas in the prefrontal cortex (FC), the hippocampus and its subregions (HPC) and the striatal cortex in the occipital cortex (OCC). For the midbrain (MB) the substantia nigra (SN) was examined (Additional file 4: Fig. S17). After the exclusion of tissue and staining artifacts, the ‘Positive Pixel Detection’ (PPD) tool was used to determine the percentage of area covered by pathological and microglia staining. The threshold values were used as follows: "downsampleFactor": 4; "gaussianSigmaMicrons": 3.0; Hematoxylin ("thresholdStain1"): 1.0; DAB ("thresholdStain2"): 0.15 (Additional file 4: Fig. S19). For automated microglia cells/mm^2^ counting the ‘Positive Cell Detection’ (PCD) tool was used (for details of the script and threshold values, see Additional file 4: Fig. S18). For the analysis of the substantia nigra, we used a script to automatically exclude dopaminergic neurons, as their natural brown coloration would interfere with the detection tools (for details of the script and threshold values, see Additional file 4: Fig. S20). All scans were initially analyzed under the same conditions. The thresholds for PPD and PCD were adjusted based on the DAB staining for individual scans. To evaluate the accuracy of software-assisted cell counting, the results of PCD and manual counting were compared (Additional file 4: Fig. S21).

To test the established semi-automated quantification method using Qupath, we analyzed the frontal cortex tissue of all cases in all four cohorts (Additional file 4: Fig. S1). A significantly large area covered by Aβ plaques and NFT was detected in AD and MIX cases compared to Control and LBD cases (Additional file 4: Fig. S1b and c). No discernible difference were seen between AD and MIX, nor in Control compared with LBD. Whereas MIX cases showed some more area covered by Aβ plaques than AD cases, this trend was reversed in MIX and AD by quantifying the phospho-tau burden. Despite the lack of statistical difference between Control and LBD, there is a tendency that more Aβ was found in LBD than in Control. However, this could no longer be observed as a function of tau. Here, no tau was detected in either group. Additional file 4: Fig. S1d shows the covered area by α-Syn in frontal cortex tissue. Only MIX cases displayed a significantly higher area covered by the protein pathology compared to the other 3 groups, while there is no difference between Control, AD and LBD cases. It should be noted that there is a tendency for AD cases to show slightly higher α-Syn detection in cortex tissue than in the LBD group.

### APOE genotyping

For APOE genotyping, DNA was extracted from FFPE and frozen brain tissue using DNA-extraction kits (QIAGEN GeneRead DNA FFPE Kit (cat. no. 180134) and QIAamp DNA Mini Kit (QIAGEN cat. no. 51304)). Using TaqMan™ SNP Genotyping Assay, human (Applied Biosystems™, Cat. no. 4351376) and TaqMan™ Genotyping Master Mix (Applied Biosystems™, Cat. no. 4371355), two single nucleotide polymorphisms (SNPs) (rs7412 and rs429358) were genotyped within the APOE gene. The relative fluorescent units (RFU) were measured by performing Real-Time—qPCR (BIO RAD CFX384 Real-Time System). The frequencies of APOE alleles and genotypes were obtained by the ratio and combination of both SNPs.

### NanoString nCounter®

Using the High Pure FFPE RNA Isolation Kit (Roche Life Science, Germany), total RNA was extracted from 20 µm section scrolls of 24 samples per brain area (HPC, FC, OCC, and MB) from FFPE blocks according to the manufacturer's instructions (https://elabdoc-prod.roche.com/eLD/web/pi/en/products/3.6.8.50.1.1). These cases were randomly selected from our cohort and included the same 6 Control, AD, MIX and LBD cases for all 4 regions of interest. RNA was then quantified using a Qubit 4 Fluorometer (Invitrogen, USA), while RNA quality assessment was performed using the Bioanalyzer 2100 (Agilent, Denmark). 5 μL of RNA at a concentration of 20 ng/mL was used for hybridization with the nCounter® Glial Subtyping Profile Panel (XT HS Glial Profiling #115,000,429) for 18 h at 65 °C (heated lid at 72 °C) (https://nanostring.com/resources/?em_resources_type=manual-instructions). Following the instructions of the nCounter® manufacturer, the hybridized product was analyzed on a NanoString nCounter® Profiler with settings for sensitive detection (fields of view (FOV) = 280). The number of times a gene was detected was tabulated in a comma separated value (CSV) format for data analysis using the nSolver version 4.0 software (nCounter® Advanced Analysis version 2.0.134). Samples were evaluated for quality control to ensure that the binding density, imaging, and detection limits of the assay were adequate. Captured transcript counts were normalized to the geometric mean of the housekeeping reference genes included in the assay and the internal positive and negative Controls of the code set [65].

### NanoString GeoMx® digital spatial profiling (DSP)

Cases for DSP were selected after IHC staining and quantification of their pathology. The sections were deparaffinized, and HIER was performed in a pressure cooker (Tintoretriever, Bio SB) for 15 min at high pressure in a 1 × Citrate Buffer pH 6.0 solution. ROIs that included Lewy body-bearing (LB) and non-Lewy body-bearing (noLB) neurons were selected for antibody profiling based on enrichment for SYTO13 (cell nuclei), MAP2 (neuronal marker), and α-Synuclein. In addition, we used the NanoString Alzheimer's Morphology Kit to assess the microenvironment around plaques (ROI Pathology) and non-plaque-bearing space (no Pathology) in AD and MIX cases. The samples were then incubated with the GeoMx® oligo-labeled primary antibodies for protein studies (Table 4) composed of: a Human Neural Cell Profiling Core, a Human Glial Cell Subtyping Panel, a Human Parkinson’s Pathology Panel, a Human Alzheimer’s Pathology Panel and a Human Alzheimer’s Extended Pathology Panel. The ROIs were selected in the cortex of frontal cortex and substantia nigra of the midbrain (Table 3). The selection of ROIs was limited to areas of gray matter in FC and substantia nigra in MB. The number of ROIs with and without pathology was kept equal for each sample and evenly distributed across samples according to the presence of pathology. For an appropriate selection of the ROIs, the DSP machine can achieve a zoom capability of up to 50 μm. To avoid too much background tissue the ROIs got a minimum diameter of 200 μm and a maximum of 205 μm. Once the ROI is chosen, the GeoMx® precisely cleaves the probes within the chosen areas, subsequently collecting each ROI in an individual well of a 96-well plate.

After loading the slides onto the DSP, an even number of ROIs were selected per slide. Oligonucleotides were collected into a 96-well plate and hybridized for 18 h at 67 °C (BIO RAD C1000 Touch Thermocycler) to fluorescent barcodes using GeoMx® Hyb Codes. After hybridization, collected samples were processed using the NanoString nCounter® for protein analyses according to the manufacturer’s instructions, generating proteomic data for each ROI for each of the selected cases [65].

### Statistical analysis

Statistical analysis was performed using GraphPad Prism 9 software (GraphPad Software). The difference between means of unpaired samples was performed using one-way analysis of variance (ANOVA) with Tukey's multiple comparisons test or an unpaired t-test as indicated. Area covered (%) or cell count (cells / mm^2^) were compared using ANOVA. Statistical significance was defined as *p < 0.05, **p < 0.01, ***p < 0.001 and ****p < 0.0001.

For spatial proteomic analysis, the GeoMx DSP data analysis tool was used with a linear mixed model (LMM) with Benjamini–Hochberg correction for multiple testing, as recommended by NanoString for handling multiple samples with repeated measurements (ROIs).

## Results

### Reduced numbers of Iba1 positive microglia in the frontal cortex of AD brain samples

**Fig. 1:**
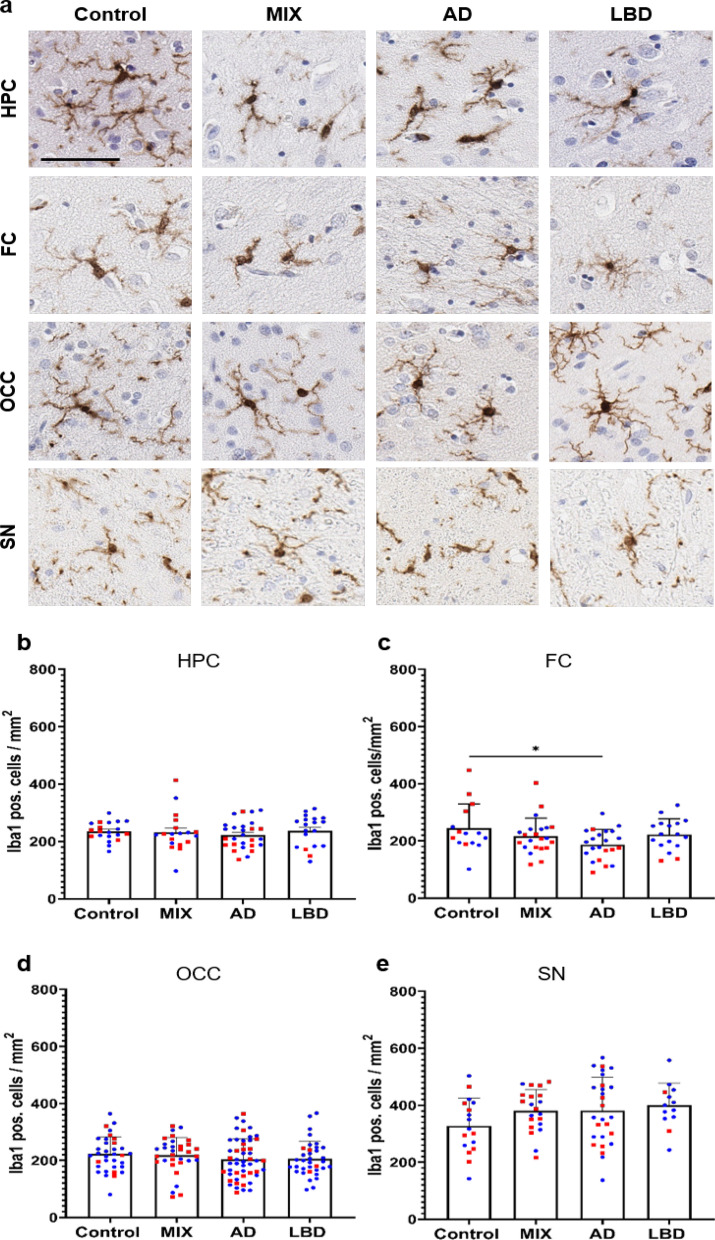
Quantification of Iba1 positive cells across different brain regions of control and disease groups. **a** Panel for immunohistochemical Iba1 staining of microglia for Control, MIX, AD and LBD cases in hippocampus (HPC), frontal cortex (FC), occipital cortex (OCC) and substantia nigra (SN). Microglia were quantified in 10 μm-thick sections stained with Iba1 antibody. **b**–**e** Quantification of Iba1 positive microglia in HPC, FC, OCC and SN. HPC data points contain hippocampal subregions (CA4-1, subiculum, entorhinal cortex, see Supp. Figure 2), OCC contains data points from subregions (striate cortex and peristriate cortex). Red – female, blue – male. No sex differences could be shown across the examined brain regions. One-way ANOVA with Tukey's multiple comparisons test was used for statistical analysis. *p < 0.05, **p < 0.01, ***p < 0.001. Scale bar = 60 μm

To profile microglia across the spectrum of ND, we selected cases with pure high ADNC (AD, n = 29), cases with pure Lewy body pathology (LBD, n = 19), and cases with mixed high ADNC and LBD (MIX, n = 22), as well as non-demented Control cases with minimal to no disease-specific protein pathology (Control, n = 23) from the UF Neuromedicine Human brain and tissue bank (see Table 1). Cases were evenly matched for sex, except for a male predominance in the LBD group, mirroring a sex bias in disease manifestation [4, 17, 58]. Age and APOE genotype were evenly distributed across groups (see Table 1). To cover brain regions affected during the progression of the ND we included in our study, we stained 10 μm thick sections of the hippocampus, a region affected early in AD, the frontal cortex, affected in intermediate stages of AD, the occipital cortex, representing late-stages of AD and the midbrain, a region affected heavily in LBD for various microglia markers. First, we used Iba1, a commonly used pan-microglia marker [30, 34]. In order to allow for high throughput and unbiased quantification of microglia numbers, we developed a protocol for automated cell detection using the open source software QuPath [5]. Initially, we compared microglia numbers from manual counting with software-generated numbers for select cases and noted a robust correlation between both datasets (Additional file 4: Fig. S21). Using our QuPath algorithm, we quantified the numbers of Iba1 positive (pos.) microglia in the aforementioned brain regions. Numbers of Iba1 pos. cells were mostly comparable between all brain regions in Control cases (Fig. 1b–e). We observed overall slightly higher numbers of Iba1 pos. microglia in the substantia nigra, consistent with previous reports [40, 80]. Although numbers of Iba1 pos. microglia were comparable between Control cases and disease conditions across almost all the analyzed brain regions, we noted that morphologically Iba1 pos. cells in the AD and MIX groups appeared to have plump and shorter processes as well as broader cytoplasm (Fig. 1a). In the frontal cortex, numbers of Iba1 pos. microglia were reduced in AD cases, while Control, MIX, and LBD cases had similar numbers of Iba1 pos. cells. This is consistent with previous reports of reduced microglia numbers in end-stage AD cases [65]. No significant differences in numbers of Iba1 pos. cells were observed in the occipital cortex, while counts in the midbrain (substantia nigra) showed a trend towards higher numbers of Iba1 pos. cells in AD, MIX, and most prominently in LBD compared to Control.

To capture different activation states of microglia matching the observed morphological differences between experimental groups, we next focused our analyses on markers associated with specific functional states of microglia.

### Increased numbers of P2RY12 positive microglia in the occipital cortex of LBD and MIX cases

**Fig. 2:**
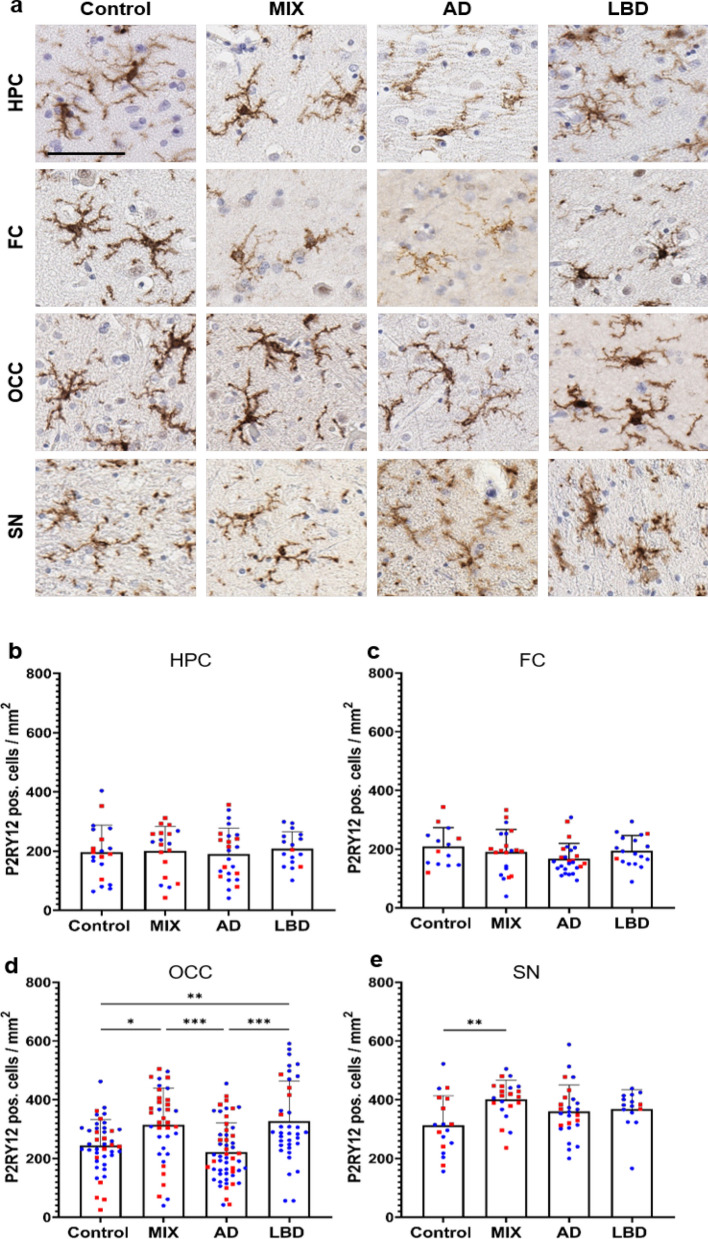
P2RY12 positive homeostatic microglia presented a unique quantification pattern in OCC. **a** Panel of homeostatic microglia which were detected using a P2RY12 antibody. P2RY12 positive microglia showed a highly ramified morphology in all regions. Quantification of P2RY12 positive cells in hippocampus (**b**), frontal cortex (**c**), occipital cortex (**d**) and substantia nigra (**e**). HPC data points contain hippocampal subregions (CA4-1, subiculum, entorhinal cortex, see Additional file 4: Fig. S3), OCC contains data points from subregions (striate cortex and peristriate cortex). Red – female, blue – male. No sex differences could be shown across the examined brain regions. One-way ANOVA with Tukey's multiple comparisons test was used for statistical analysis. *p < 0.05, **p < 0.01, ***p < 0.001. Scale bar = 60 μm

We next focused on P2RY12, which is considered a marker for homeostatic/resting microglia [19, 34, 87]. Morphologically, the majority of microglia stained with P2RY12 antibodies showed round cell bodies and more ramified processes (Fig. 2a), consistent with a resting or homeostatic state. When comparing numbers of P2RY12 pos. cells in our four experimental groups, we observed no significant difference in P2RY12 pos. cell numbers in the hippocampus and frontal cortex, although a trend towards decreased numbers of P2RY12 microglia was observed in the frontal cortex of AD cases (Fig. 2c), correlating with our observations using the pan-microglia marker Iba1 above (Fig. 1c). In the occipital cortex, however, we noted an increase in P2RY12 pos. microglia in LBD and MIX cases compared to AD and Control cases (Fig. 2d). In addition, numbers of P2RY12 pos. microglia showed a trend towards reduced numbers in AD compared to Control. In the midbrain, we observed an increase in P2RY12 pos. microglia in MIX cases compared to Control cases (Fig. 2e), while AD and LBD groups only trended towards higher P2RY12 pos. cell numbers compared to Control cases.

## Extracellular and intracellular protein pathologies drive the activation of phagocytic microglia in ND

**Fig. 3:**
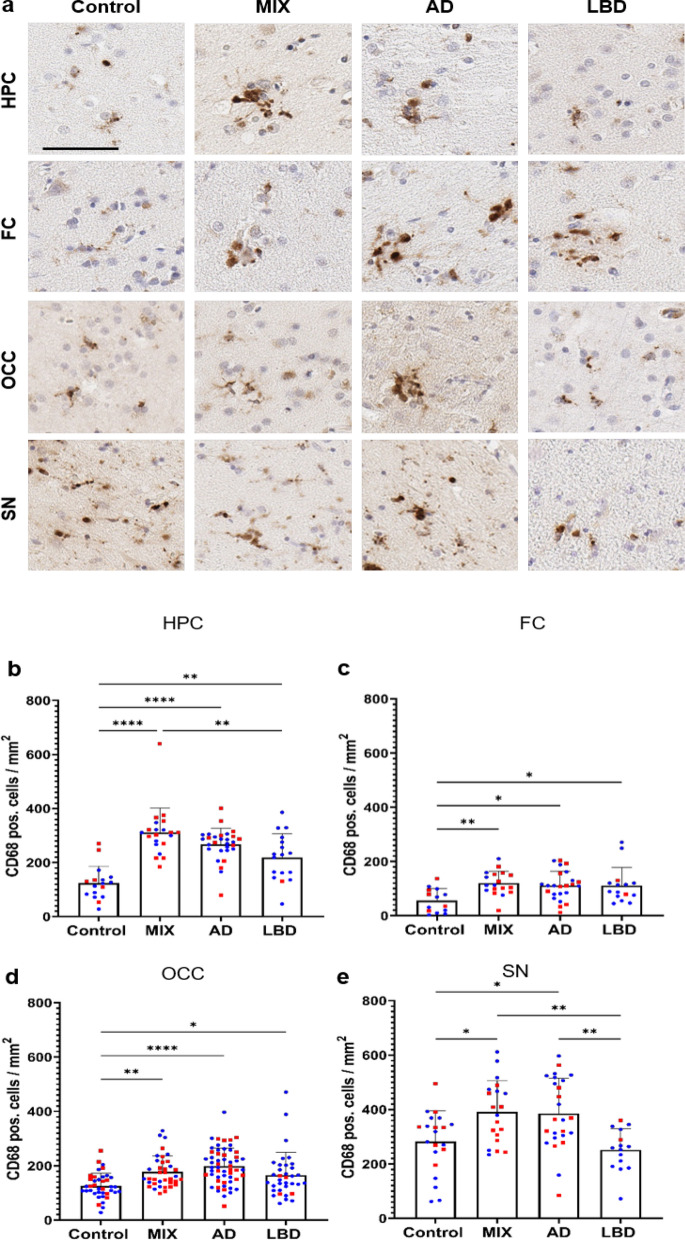
Number of detectable activated microglia are highly increased in cases with a disease background across all brain regions.** a** CD68 pos. microglia appeared in a round, amoeboid shaped morphology with single fading branches. Phagocytic active microglia start to cluster around focal events in the tissue in all disease cases. **b**–**e** Quantification revealed an increase of CD68 positive cells in disease cases compared to Controls. Major differences between disease cases were seen in HPC and SN. MIX and AD cases displayed the highest numbers in quantification (**b, e**). HPC data points contain hippocampal subregions (CA4-1, subiculum, entorhinal cortex, see Supp. Figure 4), OCC contains data points from subregions (striate cortex and peristriate cortex). Red – female, blue – male. No sex differences could be shown across the examined brain regions. One-way ANOVA with Tukey's multiple comparisons test was used for statistical analysis. *p < 0.05, **p < 0.01, ***p < 0.001. Scale bar = 60 μm

Next, we studied CD68, a lysosomal marker for macrophage linage cells primarily seen in phagocytically active microglia [24]. Morphologically, CD68 positivity was mainly detected in amoeboid microglia with round cell bodies and shortened, less complex cellular processes. While scattered CD68 pos. cells were evenly distributed throughout the tissue in Control cases, we recognized clustering of CD68 pos. cells in cases with ND-related protein pathology (Fig. 3a). In line with this observation, numbers of CD68 pos. cells were significantly increased in all disease groups (AD, MIX, LBD) compared to Control cases, across all brain regions studied. Cases exhibiting ADNC (AD and MIX) displayed the highest numbers of CD68 pos. cells, and interestingly, among those, MIX cases had slightly higher CD68 counts in the hippocampus, frontal cortex, and midbrain than pure AD cases (Fig. 3b–e).

### ADNC drive microglia dystrophy across all brain regions

**Fig. 4:**
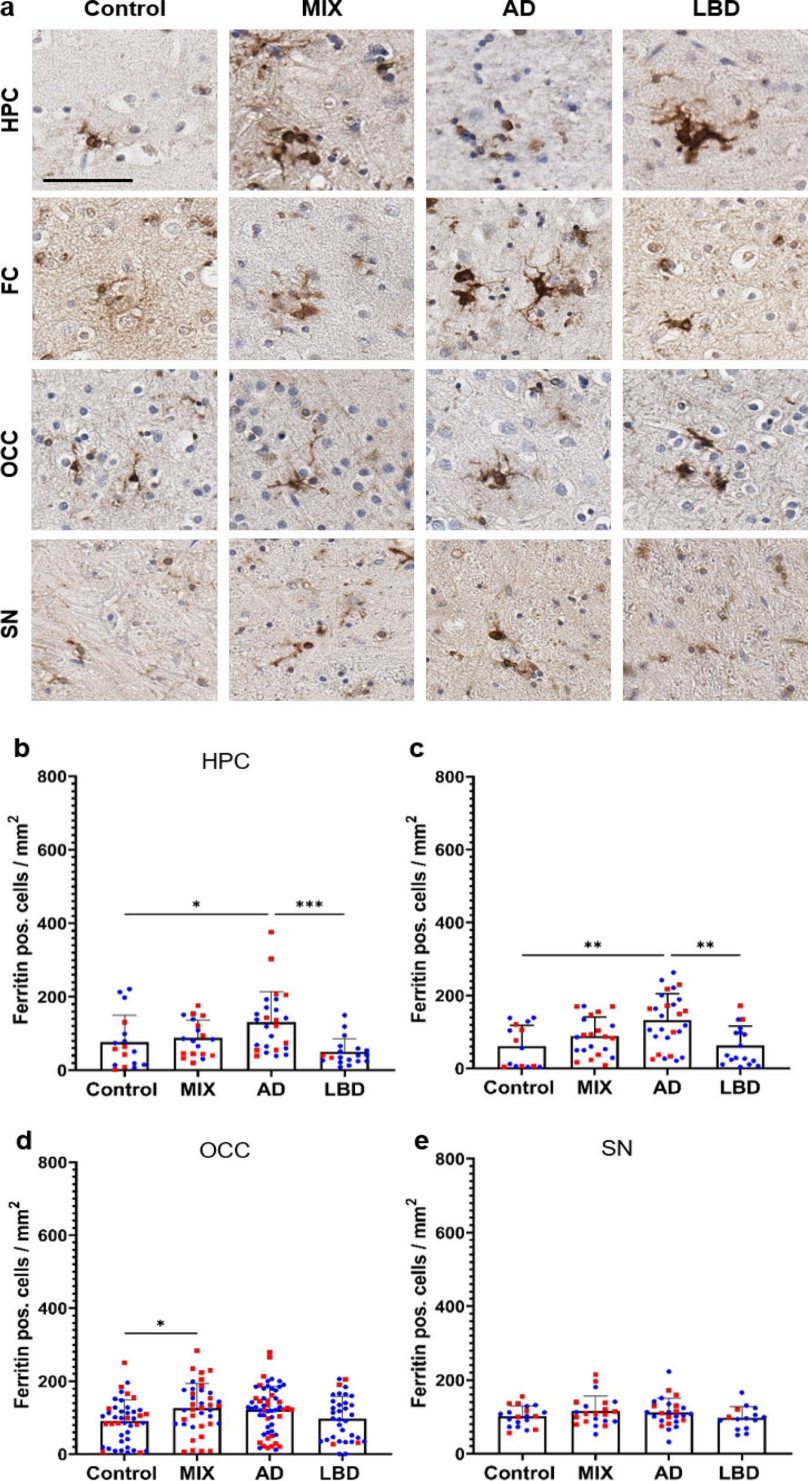
Quantification of Ferritin positive cells showed the highest amount of microglia dystrophy in cases with ADNC.** a** Overview of ferritin pos. microglia in HPC, FC, OCC and SN of control and disease cases. Quantification of ferritin pos. cells in hippocampus (**b**), frontal cortex (**c**), occipital cortex (**d**) and substantia nigra (**e**). HPC data points contain hippocampal subregions (CA4-1, subiculum, entorhinal cortex, see Supp. Figure 5), OCC contains data points from subregions (striate cortex and peristriate cortex). Red – female, blue – male. No sex differences could be shown across the examined brain regions. One-way ANOVA with Tukey's multiple comparisons test was used for statistical analysis. *p < 0.05, **p < 0.01, ***p < 0.001. Scale bar = 60 μm

Dystrophic microglia, a microglia subtype mainly found in the human brain are characterized by distorted cytoplasm, shriveled nuclei, and shortened beaded processes. Dystrophic microglia have also been noted to be positive for ferritin. Ferritin, which is expressed predominantly by neurons, microglia, and oligodendrocytes [11] is a protein that plays a crucial role in iron storage and homeostasis [22, 37]. Similar to our observations with CD68 pos. cells, scattered ferritin pos. cells in Controls were evenly distributed throughout the tissue, while ferritin pos. cells in ND cases (AD, MIX, and LBD groups) exhibited focal clustering and appeared morphologically dystrophic with shortened, beaded processes and distorted cytoplasm (Fig. 4a). Cases with ADNC (AD and MIX) exhibited the highest numbers of ferritin pos. cells in hippocampus and frontal cortex, while MIX cases showed increased ferritin pos. cell numbers in the occipital cortex (Fig. 4b–d). It is particularly noteworthy that the group with pure AD pathology had slightly higher values than the group with mixed pathology in hippocampus and frontal cortex. Cases with pure LBD pathology did not exhibit major differences in numbers of ferritin pos. cells compared to Control cases in these brain regions, suggesting that ADNC are the main driver of ferritin positivity in microglia. Interestingly, we did not observe any differences in the number of ferritin pos. cells in the midbrain between our experimental groups (Fig. 4e).

### Whole tissue transcriptomic analysis reveals robust gene expression signatures associated with ADNC, while pure LBD shows more subtle and region specific changes

**Fig. 5:**
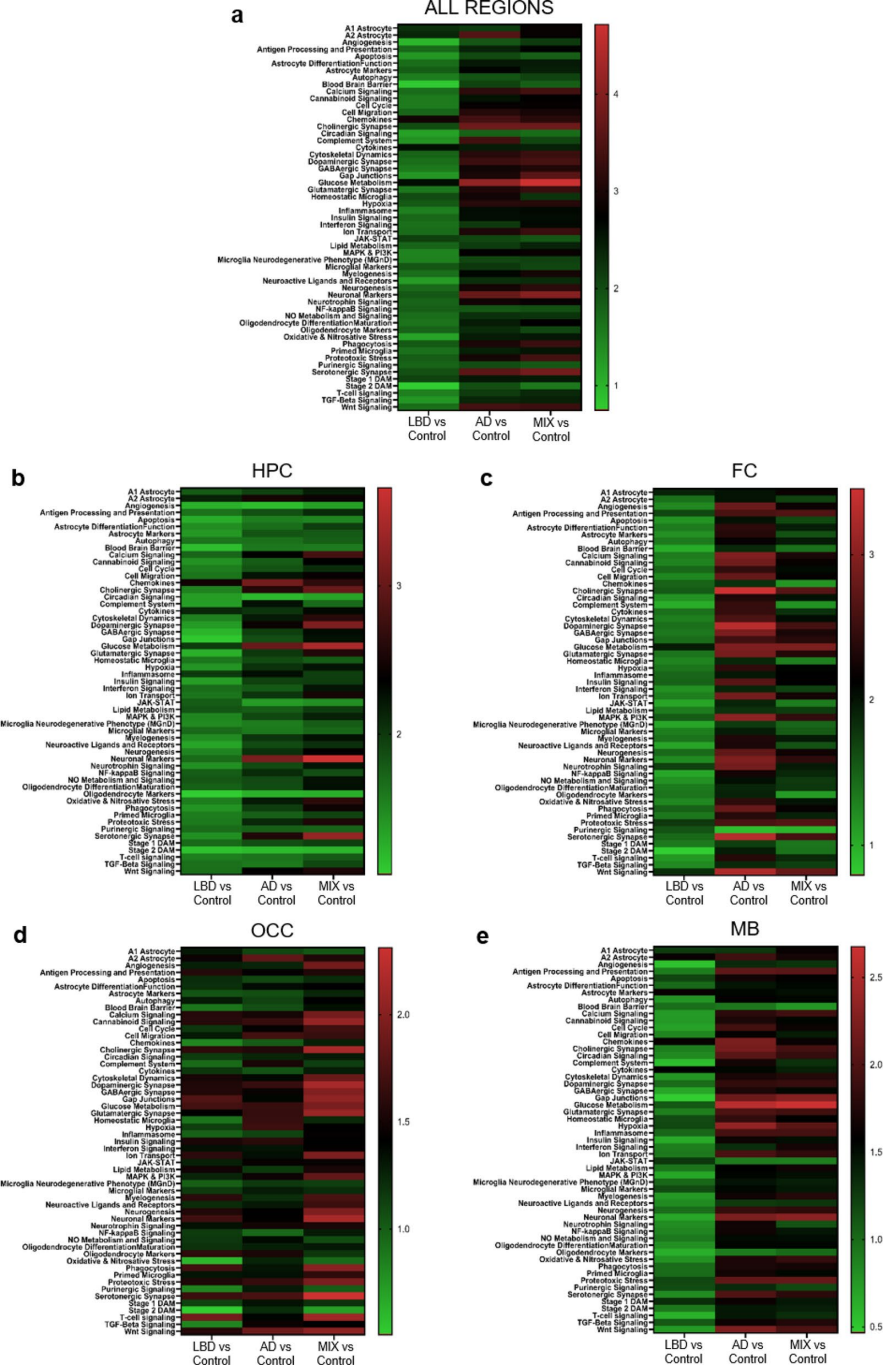
Whole tissue transcriptomic analysis across brain regions with nCounter platform. Differences in immune response patterns between AD, MIX, LBD and Control cases depend on the anatomical brain region. **a – e** Heatmap of signaling pathway scores derived from the nCounter Glial Subtype Profiling Panel for AD, MIX, LBD and Control cases independent of the brain region (**a**) and in the hippocampus (**b**), frontal cortex (**c**) occipital cortex (**d**) and midbrain (**e**). Pathway scores are depicted in alphabetical order. Green to red indicates upregulation of signaling pathway compared to Control

To get a more in-depth view of the immune response patterns across our experimental groups, we compared the expression of over 500 genes utilizing the Glial Cell Subtyping gene expression panel on the NanoString nCounter® platform in hippocampus, frontal cortex, occipital cortex, and midbrain in a representative subset of AD (n = 6), MIX (n = 6), LBD (n = 6), and Control (n = 6) cases selected from the cohort used for the histological analysis described above (Table 2, methods). RNA was extracted from consecutive sections of the same FFPE blocks used for the histological analysis (see methods section for experimental details). We performed gene set analysis (GSA) of pathway scores for the comparison of disease groups to Control in each examined region. Cases with ADNC (AD and MIX groups) showed profound and significant changes in multiple pathways when compared to Controls across all regions (Fig. 5a and Additional file 1: Table S1), while pathway changes in LBD cases compared to Control were minimal at best.

**Table 2:** Overview of sample selection for whole tissue transcriptomics with NanoString nCounter®

Region	# Samples	Disease background	Panel
HPC	24	6 Control, 6 AD, 6 MIX, 6 LBD	Glial cell subtyping gene expression
FC	24	6 Control, 6 AD, 6 MIX, 6 LBD	Glial cell subtyping gene expression
OCC	24	6 Control, 6 AD, 6 MIX, 6 LBD	Glial cell subtyping gene expression
MB	24	6 Control, 6 AD, 6 MIX, 6 LBD	Glial cell subtyping gene expression

Comparing changes in all regions, cases with ADNC (AD and MIX), displayed overall a similar up- and downregulation of signaling pathways, while LBD cases tended to show a higher similarity to Control cases (Fig. 5a). Notably, both AD and MIX cases showed a strong upregulation in synaptic pathways and glucose metabolism, while microglia-related pathways like Stage 1 DAM, Homeostatic Microglia, or Primed Microglia were only slightly increased. In contrast to LBD and MIX, AD cases displayed a more prominent increase in complement system pathways (Fig. 5a).

Comparing whole tissue gene expression profiles in the hippocampus, a region affected early in AD, we noted an expected upregulation of multiple pathways including neuronal marker pathways and Glucose metabolism, as well as microglial Stage 1 DAM in AD and MIX cases, while the examined pathways were comparable between LBD and Controls (Fig. 5b).

In the frontal cortex, multiple pathways were strongly upregulated in the AD group, including synaptic and neuronal pathways, Glucose metabolism and MAPK & PI3K signaling as well as Phagocytosis. MIX cases shared some of the upregulated pathways, including Glucose metabolism and synaptic pathways (Fig. 5c), while LBD cases were again more comparable to Control cases.

While AD showed a more prominent upregulation of signaling pathways in HPC and FC (Fig. 5b and c), the trend was shifted towards MIX cases in the occipital cortex (Fig. 5d). Cases with a pure protein pathology (AD and LBD) showed a similar expression pattern of genes, while MIX cases trended to show an upregulation of neuronal and synaptic pathways. LBD cases showed a more general increase in inflammation-related pathways compared to Controls, while AD cases displayed a more specific activation of the immune system reflected by pathways such as Stage 1 and 2 DAM, Primed and Homeostatic microglia.

Finally, we analyzed whole tissue transcriptomic changes for pathways covered in the Glial Cell Profiling Panel in the midbrain, a region that is mainly affected by LB pathology (Fig. 5e). Interestingly, we observed similar pathway expression patterns as in the hippocampus and frontal cortex. Cases with ADNC (AD and MIX) shared upregulated pathways, including Glucose metabolism, synaptic pathways, antigen presentation, Phagocytosis and Primed microglia, but we noted a slight increase in the expression of Stage 1 DAM-related genes in AD cases. LBD cases were again very similar in the expression patterns of the examined pathways compared to Controls. We noted however a slight upregulation of astrocyte marker, cytokines and microglial Stage 1 DAM pathways in LBD cases compared to Controls, supporting the notion of a brain region-specific and more subtle activation of the local immune system in LBD cases compared to cases with ADNC (AD and MIX groups).

### Spatial proteomics highlights a strong local immune response to ADNC, while the response to pure LBD is region and disease specific

**Fig. 6:**
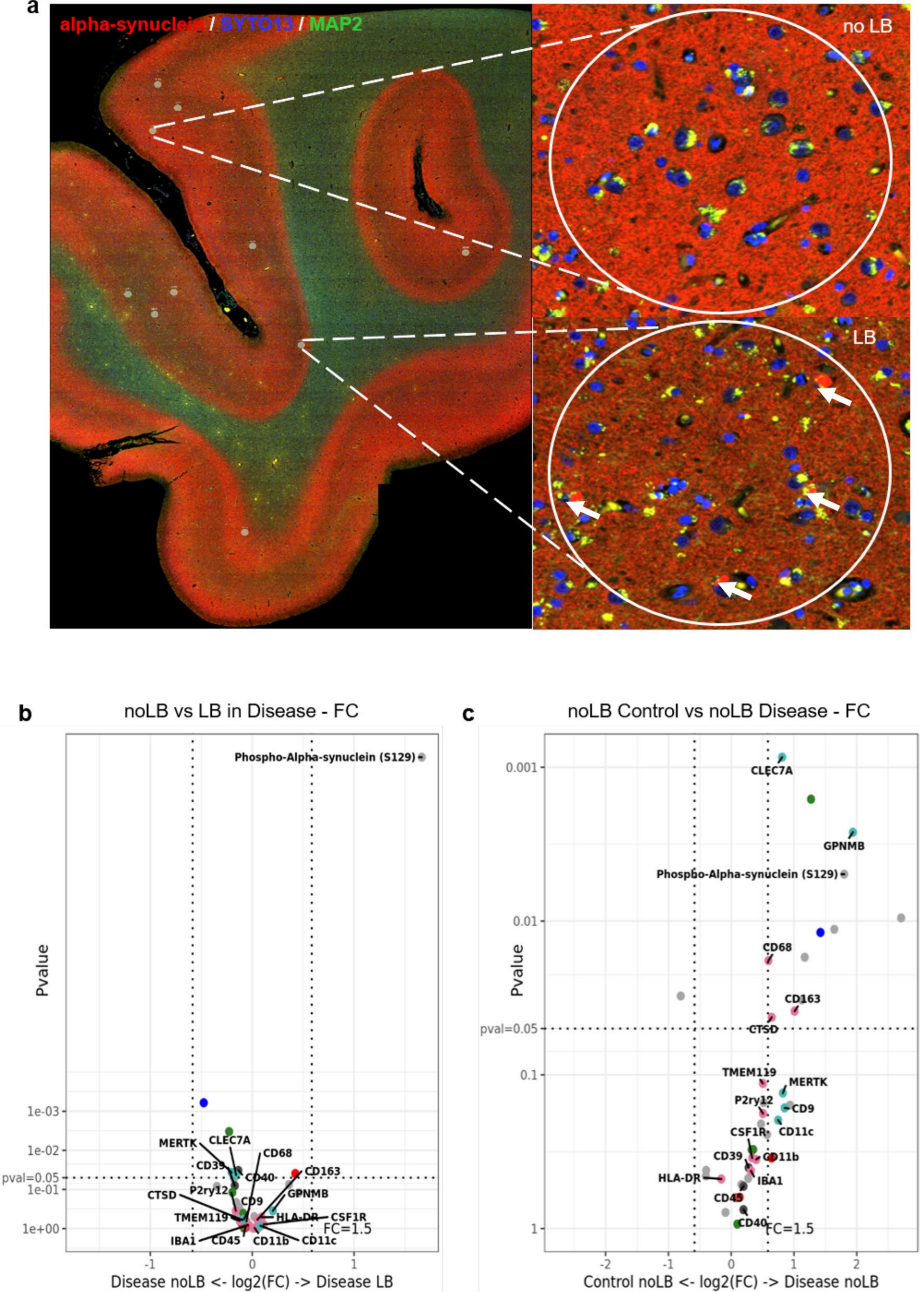
Digital spatial proteomic analysis of cases with Lewy body pathology and Controls in frontal cortex. **a** Selected region of interests (ROIs) from tissue with Lewy body pathology (MIX, LBD) and Control. For the selection of pathology, circles of 200 μm were drawn around Lewy body bearing neurons and neurons without pathology. Morphology markers α-Syn (NanoString α-Syn Alexa Fluor® 594) (red) and α-MAP-2 (NanoString Alexa Flour® 532) (green) were used to distinguish neurons with accumulations of α-Syn in form of Lewy bodies (arrows). DNA was stained with SYTO13 dye (blue). **b** Volcano plot for the expressed proteins in cases with Lewy body pathology (MIX and LBD), comparison of the microenvironment of Lewy body bearing neurons and neurons without pathology. **c** Volcano plot for the expressed proteins in the microenvironment of non-Lewy body bearing neurons in disease (MIX and LBD) and Control cases. In volcano plots, unadjusted *p*‐value of 0.05 and fold‐change (FC) of 1.5 were used to identify differentially expressed proteins. Color code of detected proteins based on used NanoString Antibody Panels: blue – AD, red – Astrocyte/Inflammation, green – PD, pink – Microglia, turquoise – Disease-Associated Microglia, dark grey – Microglia/Inflammation, light grey – other

To get a more detailed picture of the local proteomic environment around pathological protein aggregates in AD, MIX and LBD we next used the NanoString GeoMx® digital spatial profiler (DSP) platform. First, we investigated the proteomic microenvironment around Lewy body-bearing neurons, in samples of LBD and MIX in the FC (n = 23, 182 ROIs, Table 3) (Fig. 6a). For the selection of regions of interest (ROI) we used double staining with an α-Syn antibody (NanoString α-Syn Alexa Fluor® 594) and a α-MAP-2 (NanoString Alexa Flour® 532) to select accumulations of α-Syn in form of Lewy bodies-in neuronal somas in cortical regions (Fig. 6a, arrows). Map2 staining was used to select neurons without Lewy body pathology (Fig. 6a). We selected 182 ROI with a diameter of 200–205 μm each, encompassing neurons harboring Lewy bodies (LB, Fig. 6a) or neurons without significant Lewy body pathology (noLB, Fig. 6a). In addition, we collected neurons without Lewy body pathology (noLB) ROIs from Control cortical tissue. Comparing the local microenvironment between LB and noLB in the disease groups (LBD and MIX), we did not observe major changes in the expression of 40 targets contained in the panels we used for our study (Parkinson’s Pathology Panel, Neural Cell Profiling Panel, and Glia Cell Subtyping Panel, see Additional file 2: Table S2 for details), except for a strong upregulation of phospho-α-Syn (S129) in the LB ROI, confirming successful selection of pathology bearing vs. non-pathology bearing neurons (Fig. 6b, also see Additional file 3: Table S3). Notably, we did not observe major changes in the expression of microglia-related markers around LB neurons, compared to noLB neurons in the disease groups, suggesting that Lewy body pathology does not induce strong changes in the local proteomic environment. Comparing noLB ROI from the disease group with noLB ROI from control tissue however revealed increases in specific activated microglia markers including CLEC7A, GPNMB [70] and CD68 in disease tissue (Fig. 6c), supporting the notion of a disease-specific, global immune activation in LBD and MIX cases.

**Table 3:** Overview of sample and ROI selection for spatial profiling with NanoString GeoMx®

Region	Panel	# Scans/Samples	# ROIs	ROI labels	Disease background
FC	Parkinson’s morphology	23	182	noLB, LB	3 Control, 8 MIX, 4 AD, 8 LBD
MB	Parkinson’s morphology	20	226	noLB, LB	3 Control, 8 MIX, 9 LBD
FC	Alzheimer’s and Parkinson’s morphology	12	192	Plaque, Plaque with LB, noLB, LB	4 Control, 4 AD, 4 MIX

**Fig. 7:**
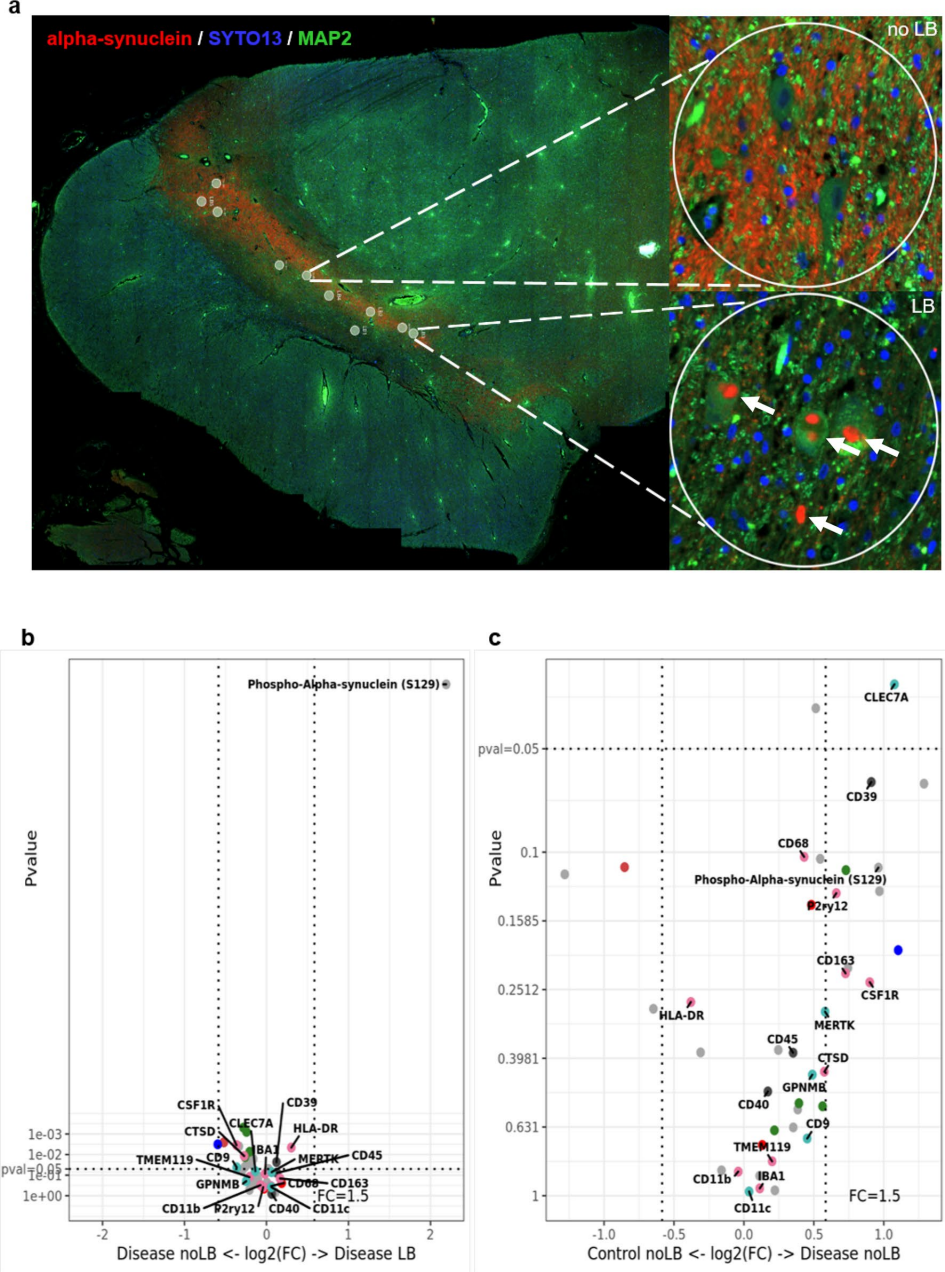
Digital spatial profiling of the microenvironment around Lewy body and non-bearing neurons of cases with Lewy body pathology and Controls in midbrain. **a** Selected region of interests (ROIs) from tissue with Lewy body pathology (MIX, LBD) and Control. For the selection of pathology, circles of 200 μm were drawn around Lewy body bearing neurons and neurons without pathology. Morphology markers α-Syn (NanoString α-Syn Alexa Fluor® 594) (red) and α-MAP-2 (NanoString Alexa Flour® 532) (green) were used to distinguish neurons with accumulations of α-Syn in form of Lewy bodies (arrows). DNA was stained with SYTO13 dye (blue). **b** Volcano plot for the expressed proteins in cases with Lewy body pathology (MIX and LBD), comparison of the microenvironment of Lewy body bearing neurons and neurons without pathology. **c** Volcano plot for the expressed proteins in the microenvironment of non-Lewy body bearing neurons in disease (MIX and LBD) and Control cases. In volcano plots, unadjusted p‐value of 0.05 and fold‐change (FC) of 1.5 were used to identify differentially expressed proteins. Color code of detected proteins based on used NanoString Antibody Panels: blue – AD, red – Astrocyte/Inflammation, green – PD, pink – Microglia, turquoise – Disease-Associated Microglia, dark grey – Microglia/Inflammation, light grey – other

Next, we examined neurons in the substantia nigra from LBD, MIX and Control cases (n = 20, 226 ROIs, Table 3). Similar to our approach in frontal cortex samples, we used α-Syn and α-MAP-2 double staining to highlight LB-bearing and noLB neurons. We selected 226 ROIs (200 μm diameter) around Lewy body bearing neurons (LB) and neurons without pathological α-Syn accumulation (noLB) in LBD and MIX cases. As baseline, we used ROIs collected from neurons in the substantia nigra of Control tissue (noLB). In all ROI we quantified the expression levels of markers from the following NanoString GeoMx® antibody panels: Parkinson’s Pathology Panel, Neural Cell Profiling Panel, and Glia Cell Subtyping Panel (Table 3, see Additional file 2: Table S3 for details). Similar to our observations in frontal cortex tissue, we did not see major local changes in immune-related proteins around LB-bearing neurons (Fig. 7b, also see Additional file 3: Table S3) when comparing ROI from the MIX and LB groups. A strong upregulation of phospho-α-Syn (S129) in ROIs with Lewy bodies again validated our ROI selection procedure. When comparing noLB ROIs from Controls and disease tissue however, we observed an upregulation of innate immune system markers including CLEC7A, CD68, and CD39 [31] (Fig. 7c) in MIX and LB cases, supporting the notion of a more global disease-driven immune response over a local inclusion-driven immune response.

**Table 4:** Antibodies used for IHC and deep spatial profiling

Antibody	Target	Host	Dilution	Company
*IHC*				
Iba1	Iba1	rabbit	1:8000	Abcam
P2RY12	P2RY12	rabbit	1:1000	Merck/Millipore Sigma (HPA014518)
Ferritin	Ferritin	rabbit	1:1000	Merck/Millipore Sigma (F6136-1MG)
CD68	CD68	rabbit	1:2000	ThermoFisher SCIENTIFIC (PA578996)
Ab5	Aβ plaques	mouse	1:1000	[41]
AT8	Phospho-tau	mouse	1:5000	Invitrogen (MN1020)
94-3A10	α-Synuclein	mouse	1:10,000	[73]
*DSP*				
GeoMx® Parkinson’s Morphology Kit	MAP-2 & α-Synuclein		1:40	NanoString® (121,300,307)
Parkinson’s pathology panel	10 targets for human PD pathology		1:25	NanoString® (121,300,110)
Glial cell subtyping	10 targets for human glial cell subtyping		1:25	NanoString® (121,300,116)
Neuronal cell profiling	20 targets for human neural cell profiling		1:25	NanoString® (121,300,108)
Beta-Amyloid (D54D2)	Aβ plaques		1:40	Cell signaling technology (35363S)
Alzheimer’s pathology panel	10 targets for human AD pathology		1:25	NanoString® (121,300,109)
Alzheimer’s extended pathology panel	10 targets for human AD pathology		1:25	NanoString® (121,300,114)

**Fig. 8:**
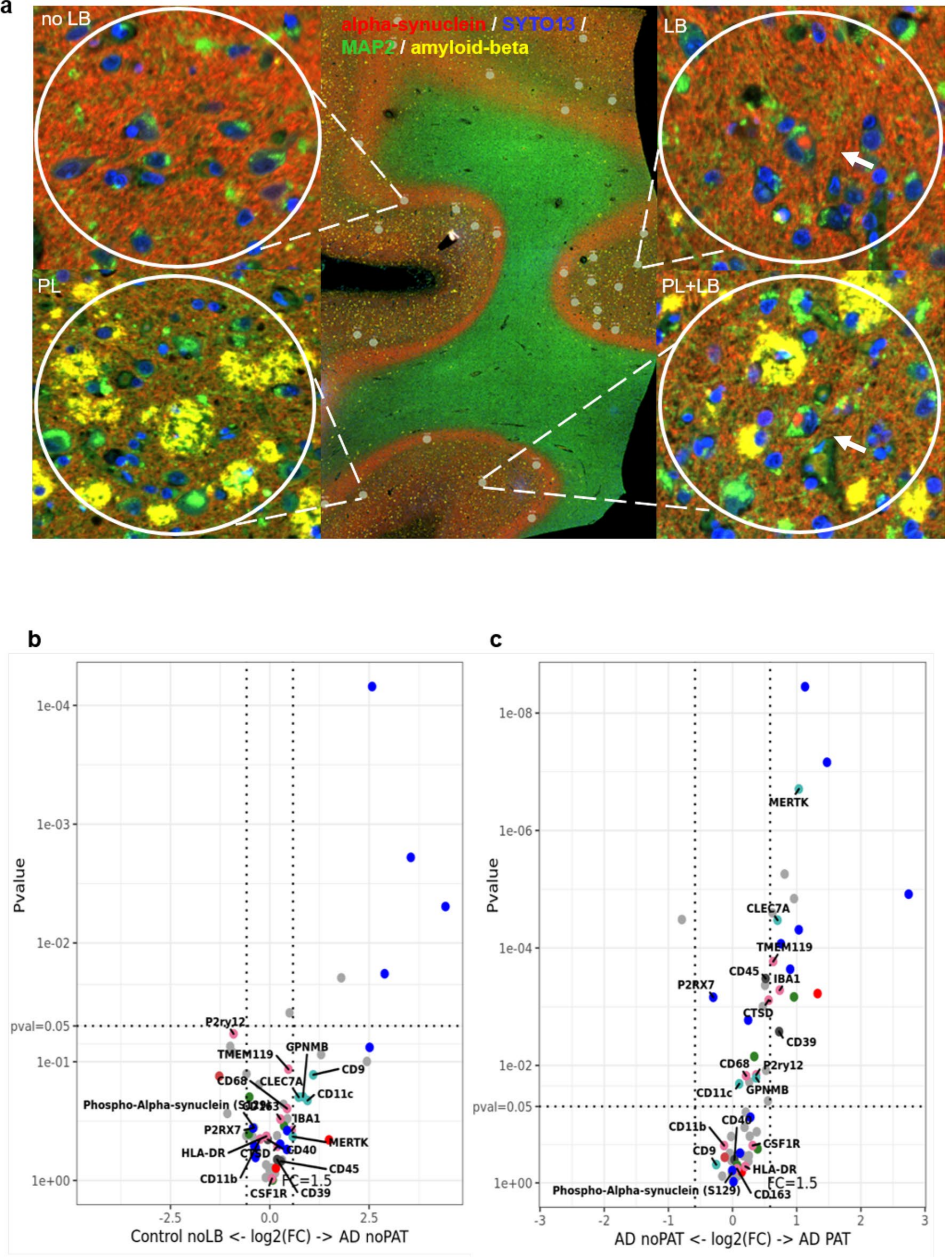
Analysis of microenvironment around neurons with Aβ plaque pathology in cases with ADNC (AD and MIX) and Controls in frontal cortex. **a** Selected region of interests (ROIs) from tissue with ADNC (AD, MIX) and Control. For the selection of pathology, circles of 200 μm were drawn around neurons with and without Aβ plaque. Morphology markers α-Syn (NanoString α-Syn Alexa Fluor® 594) (red), α-MAP-2 (NanoString Alexa Flour® 532) (green) and beta-amyloid (D54D2) (Alexa (R) 647) (yellow) were used to distinguish neurons with accumulations of α-Syn in form of Lewy bodies (arrows) and Aβ plaques. DNA was stained with SYTO13 dye (blue). **b** Volcano plot for the expressed proteins around neurons without pathology in cases with Aβ pathology (AD, MIX) and Controls. **c** Volcano plot for the expressed proteins around neurons with and without pathology in disease cases (AD, MIX), comparison of the microenvironment of pathology and non-bearing neurons within the same tissue. In volcano plots, unadjusted p‐value of 0.05 and fold‐change (FC) of 1.5 were used to identify differentially expressed proteins. Color code of detected proteins based on used NanoString Antibody Panels: blue – AD, red – Astrocyte/Inflammation, green – PD, pink – Microglia, turquoise – Disease-Associated Microglia, dark grey – Microglia/Inflammation, light grey – other

After profiling the microenvironment of neurons with pure intracellular LB pathology suggested a more global, disease-specific response, and since our histological analysis pointed to an important role of ADNC in driving microglia activation and microglia dystrophy, we next examined the response around extracellular Aβ plaques in MIX and AD cases. For this analysis we added additional protein panels (Alzheimer’s Pathology Panel and Alzheimer’s Pathology Extended Panel, see Additional file 2: Table S3 for details) to the panels used for the above described comparisons, thus increasing the number of profiled antibodies up to 60, including different tau species, Aβ 1-40 and 1-42 and Tdp-43. This enabled us to better differentiate the immune response to extracellular (Aβ), intracellular (LB), and mixed pathologies (Aβ and LBs) (n = 12, 192 ROIs, Table 3). Focusing on cases with ADNC, we selected the ROIs of 200 μm in diameter around Aβ plaques, which were labeled with a beta-amyloid (D54D2) (Alexa (R) 647) antibody in AD and MIX cases (Fig. 8a). We compared areas with plaques and plaque free ROIs from the same cases and plaque free ROI from Controls (n = 12, 192 ROIs, Table 3). Comparing plaque free ROI in Control and AD cases we saw a subtle upregulation of microglial DAM proteins like CD9, CD11c, and CLEC7A in AD tissue, similar to our observations of a global, disease-specific immune activation in LBD tissue. In addition, we noted decreased expression of P2RY12 in AD (Fig. 8b, also see Additional file 3: Table S3), which is consistent with the results of our histological analysis and reports that AD pathology induces a reduction of homeostatic microglia markers [65, 72]. Comparing plaque and no plaque ROI in AD tissue, however, in contrast to our observation in LBD tissue, we identified a very pronounced local upregulation of immune-related markers around Aβ plaques (plaque) compared to ROIs without pathology (no plaque, Fig. 8c). Notably, we noted increased expression of DAM protein markers, including MERTK, CLEC7A, and GPNMB, and also observed stronger signals of other microglia-associated markers including IBA1, TMEM119, and CD68 around Aβ plaques.

## Discussion

In this study, we used a large cohort of human postmortem brains to investigate the local immune response around extra- and intracellular protein pathologies in AD and LBD. We identified a strong, local immune activation pattern in cases with ADNC, regardless of the presence or absence of Lewy body co-pathology (AD and MIX cases), while cases with pure LB pathology showed a more attenuated, global, disease-related immune activation signature. Specifically, activated and dystrophic microglia phenotypes were strongly associated with the presence of extracellular Aβ pathology in cases with ADNC.

In the first part of this study, we focused on the immunohistochemical quantification of different states of microglia (homeostatic, activated, and dystrophic) in brain regions, that are important for the progression of ND (AD and LBD). Previous studies, which mostly focus on individual brain regions, have demonstrated that in the early stages of ND, such as AD or PD, microglia exhibit a specific activation pattern [14, 26, 62, 64, 75]. While there is a large body of literature on microglia activation in AD, there is a lack of studies examining immune activity in LBD. In our study, we found that regardless of the disease or the brain region examined, there were no significant differences in the numbers of Iba1 pos. microglia. Only a slight reduction of Iba1 pos. cells was observed in cases with pure ADNC. While previous studies have reported that the number of Iba1 pos. cells is reduced in high AD cases [42, 53, 65], we did not observe a decrease in cell numbers in cases with LBD pathology (MIX and LBD). Since P2RY12 is often co-localized with Iba1 [87], it is less surprising that we observed a similar expression pattern of these two microglia markers, with a slight reduction of positive numbers in AD cases in HPC and FC. While Iba1 is considered a pan microglia marker, which can be found across all functional microglia states (activated, homeostatic, dystrophic, etc.), P2RY12 is often used as a marker for homeostatic microglia. P2RY12 is important for chemotaxis of microglia (ATP detection), neuron maintenance, and synaptic pruning [19], however, the exact function of this purinergic receptor remains unclear. Interestingly, we noted increased numbers of P2RY12 pos. cells in OCC of cases with LB pathology (MIX and LBD). The visual cortex is considered a brain region with high P2RY12 activity, since visual impacts cause an increase of neuron-microglia interaction [90]. Homeostatic microglia interact with dendritic spines of visual V1 neurons and help to maintain synaptic connections in that area [85]. ND with Lewy bodies are reported to experience well-formed recurrent complex visual hallucinations and visuoperceptual deficits which come with hyperactivation of neurons and neurotransmitter imbalance [36, 81]. The increased neuronal activity in the OCC is thought to cause increased microglia recruitment to maintain neuron health and synapses [16, 90]. In strong contrast, when studying markers associated with microglia activation, we observed a marked increase in activated/phagocytic and dystrophic cells in all tissue samples with ND, especially in the pure AD and MIX cases. This is also consistent with the current literature, which reports increased activation of microglia in the affected brain areas of individuals with AD [3, 65, 84, 92]. Interestingly, our study shows that the largest increase in activated microglia can be observed in HPC and FC. An increase in CD68- and Ferritin pos. cells was noted especially in cases with ADNC (AD and MIX). It seems that these areas, which are affected early in AD, show the greatest extent of chronic microglia activation and dystrophy, while in OCC and MB, the differences between disease groups (AD, MIX and LBD) are less pronounced compared to controls. Ferritin is a microglial marker for dystrophic cells [45] and is also described as a marker for chronically activated microglial cells [33]. Since it can also be associated as a marker for senescence, it was to be expected that Ferritin levels would be elevated in aging brains [43, 47, 49]. However, the results of this study are more in line with the current literature, which cites Ferritin as a marker of microglial cell death in ND [32, 45, 57, 76]. The reduction in Iba1 pos. and homeostatic cells in ADNC cases and the increase in activated and dystrophic microglial cells may be related to the fact that microglial cells undergo a much greater phenotypic shift in response to ADNC than in response to normal aging-related changes or in cases with pure intracellular pathology (e.g. LBs). While this change is less pronounced in control subjects or LBD, microglia tend to become stuck in an activated state around extracellular pathology (Aβ plaques) and enter a "burned-out" state, which leads to increased death and dystrophy later in the course of the disease [45, 79].

Since our IHC data suggested a direct link between microglia activation and microglia dystrophy with extracellular pathology, we next thought to get a more comprehensive profile of microglia activation using whole tissue transcriptomic analysis.

Using the NanoString nCounter® technology, we analyzed glial cell related signaling pathways in a subset of cases from our histological analysis. Overall, this analysis revealed that in ND cases the entire brain is in a state of general immune activation, regardless of the underlying specific disease process. We noted that the signaling pathway profiles of AD and MIX cases were more similar to each other than those of LBD cases or controls. Previous studies have shown that AD cases exhibit impairment in glucose metabolism and reduced glucose uptake [18, 28, 39]. Our data support this notion but also show that dysregulation of glucose metabolism is not only observed in pure AD cases but also in cases with mixed pathology (MIX). Interestingly, the upregulation of microglia signaling pathways we observed by whole tissue transcriptomics, was less pronounced than expected from the data of our histological analysis. The specific pathways appeared to be dependent on the brain region as well as the underlying disease condition. In HPC, FC and MB cases with ADNC (AD and MIX groups) were more similar than LBD or controls. The differences between experimental groups were smallest in the hippocampus region (comparison of Stage 1 and 2 DAM, MGnD or Primed microglia) than in FC or MB, where we observed a much stronger activation of microglia in AD and MIX than in LBD. The most profound upregulation of immune response pathways however was noted in the OCC. This could reflect more early disease changes, since the OCC is affected late in AD [65]. MIX cases showed the most pronounced upregulation of synaptic signaling pathways, glucose metabolism, or neuronal markers. In comparison, AD cases showed a more prominent and specific upregulation of microglial markers such as Stage 1 and 2 DAM, Primed microglia, and Complement system.

The whole tissue transcriptomic results overall showed a less pronounced activation of microglia pathways in cases with extracellular protein pathologies (AD and MIX) as expected from our immunohistochemical analysis of microglial activation markers. This suggest that there is not a general, tissue-wide activation of microglia, but a focal, pathology-driven event in response to extracellular Aβ plaques.

To confirm this hypothesis and to highlight the focal events around different intracellular and extracellular protein aggregates, we decided to study the local changes with selected protein markers using the NanoString Digital Spatial Profiling platform.

On the NanoString GeoMx® Digital Spatial Profiling platform, we investigated the tissue microenvironment around specific protein pathologies in the last part of this study. By targeting specific ROIs, we were able to perform limited proteomic analyses around disease-specific protein pathologies, such as Lewy bodies or Aβ plaques and investigate specific proteins associated with different microglia activation states in this local environment. Interestingly, when comparing Lewy body-bearing neurons between disease groups (LBD and MIX), we observed no significant increase in microglia or inflammatory markers, albeit a significant increase of phospho-α-Syn (S129) in LB-bearing neurons demonstrated successful selection of the respective ROI. When comparing noLB tissue from disease tissue with noLB tissue from control cases we observed a robust upregulation of microglia activation-associated markers in the disease group. This suggests that the immune activation in cases with intracellular LB pathology is more global, disease-specific than locally concentrated around LB-bearing neurons. This contrasts with the data from the comparison of AD and MIX cases. In the local microenvironment around extracellular Aβ plaques, we identified a significant upregulation of markers for activated and DAM microglia such as MERTK, CLEC7A or GPNMB. The spatial proteomic dataset confirms our histological data, where we noted that activated and dystrophic microglia phenotypes were strongly associated with the presence of extracellular Aβ pathology in cases with ADNC, while cases with pure LB pathology showed a more attenuated, global disease-related immune activation signature.

Our study has several limitations. Although we have made our best efforts to create a cohort of brains of the same age that are balanced in terms of sex, pathology, and APOE genotype, we are dependent on the availability of postmortem tissue. This was most apparent in the sex bias of LBD cases, which are known to be more commonly seen in men, whereas AD is more common in women [17, 58, 61, 71]. Another limitation of this study is that we are studying postmortem human tissue. This represents only the endpoint of the disease and reflects only a short period of disease progression. In particular, the interpretation of our data with respect to a potential change in microglia phenotype during disease progression can only be inferred from cross-sectional analysis and comparison of different brain regions. Longitudinal assessment of microglia activation during disease progression in one person would be desirable, but is not achievable with currently available in vivo microglia markers.

Overall, we conclude that there is a significant correlation between microglial activation and the presence of extracellular protein pathologies, while intracellular pathologies drive a more subtle and more global immune response. Furthermore, there are important brain region differences in the observed microglia and immune activation phenotypes. Our study underscores the complexity of the immune response towards ND-related protein pathologies in human brains and highlights the importance of analysis of post-mortem brains for successful translation of in vitro and in vivo experimental studies.

### Supplementary Information

**Additional file 1: Table S1.**

**Additional file 2: Table S2.**

**Additional file 3: Table S3.**

**Additional file 4: Figure S1–S21.**

## Abbreviations

AD: Alzheimer’s disease
ADNC: Alzheimer’s disease neuropathological changes
ADRD: Alzheimer’s disease related dementias
APOE: Apolipoprotein E
Aβ: Amyloid beta
CD68: Cluster of Differentiation 68
CERAD: Consortium to establish a registry for Alzheimer's disease
FC: Frontal cortex
GSA: Gene set analysis
HPC: Hippocampus
Iba1: Ionized calcium-binding adapter molecule 1
LATE: Limbic-predominant age-related TDP-43 encephalopathy
LB: Lewy body
LBD: Lewy body diseases
MB: Midbrain
MGnD: Microglia Neurodegenerative Phenotype
MIX: Cases with mixed pathologies
ND: Neurodegenerative disease
NFT: Neurofibrillary tangles
noLB: Non-Lewy body bearing neurons
noPAT: Microenvironment without pathology
OCC: Occipital cortex
P2RY12: Purinergic receptor P2Y12
PART: Primary age-related tauopathy
PAT: Microenvironment with pathology
PD: Parkinson’s disease
Pos.: Positive
SN: Substantia nigra
α-Syn: α-Synuclein
