# Supplementary material for: Disease and brain region specific immune response profiles in neurodegenerative diseases with pure and mixed protein pathologies
Source: Acta Neuropathol Commun. 2024 Apr 5;12:54. doi: 10.1186/s40478-024-01770-7 (PMC10996248; doi:10.1186/s40478-024-01770-7)
Supplement: Supplementary file 4 — Additional file 4: Figure S1–S21. [file 40478_2024_1770_MOESM4_ESM.docx]

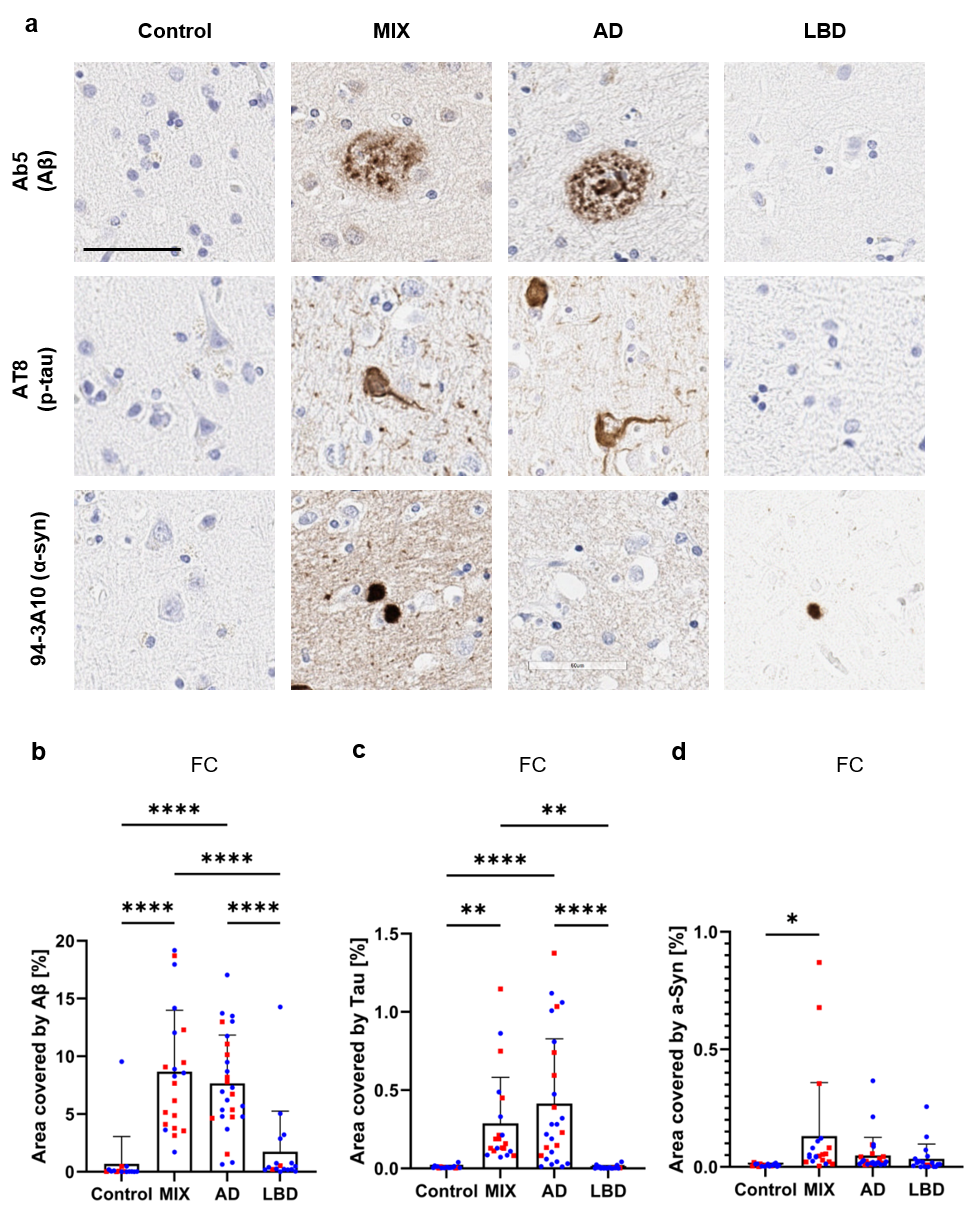


**Supplementary figure S1.** **Quantification of protein pathologies in the frontal cortex. a** – Overview of IHC stained protein pathologies in frontal cortex. Antibodies used: Ab5 – Aβ plaques, AT8 – phospho-tau, 94-3A10 – α-Syn. **b – d** Quantification of protein pathologies using QuPath algorithm to verify case selection depending on their neuropathological scoring. **b** – Area covered by Aβ plaques, **c** – area covered by phospho-tau, **d** – area covered by α-Syn.


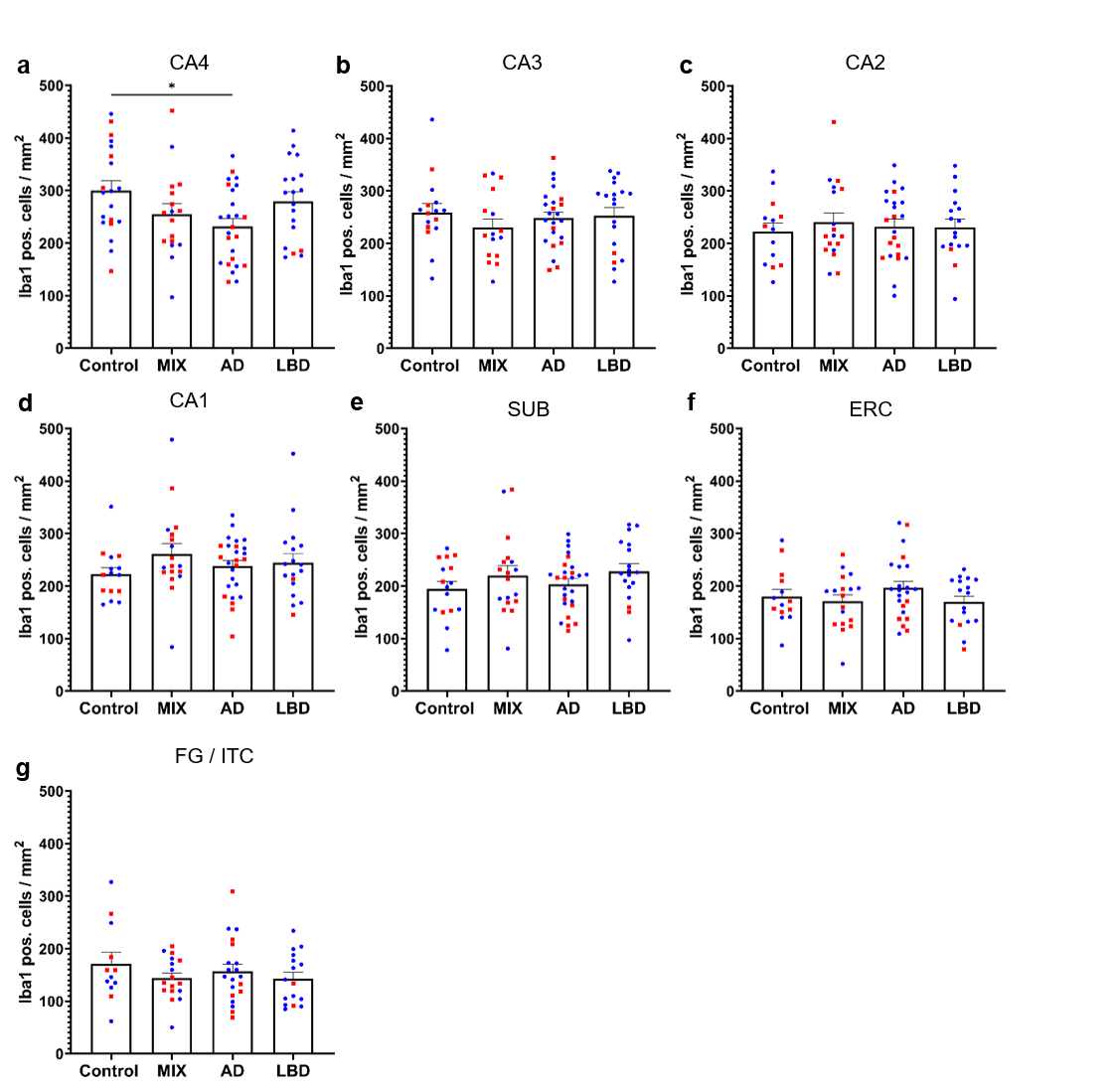


**Supplementary figure S2.** **Quantification of Iba1 pos. cell count in hippocampal subregions. a** – **g** Overview of Iba1 pos. stained microglia in CA (cornu ammonis) 4 -1, SUB (subiculum), ERC (entorhinal cortex), FG/ITC (fusiform gyrus / inferior temporal cortex). **Red** – female, **blue** – male. No sex differences could be shown across the examined brain regions. One-way ANOVA with Tukey's multiple comparisons test was used for statistical analysis. *p < 0.05, **p < 0.01, ***p < 0.001.


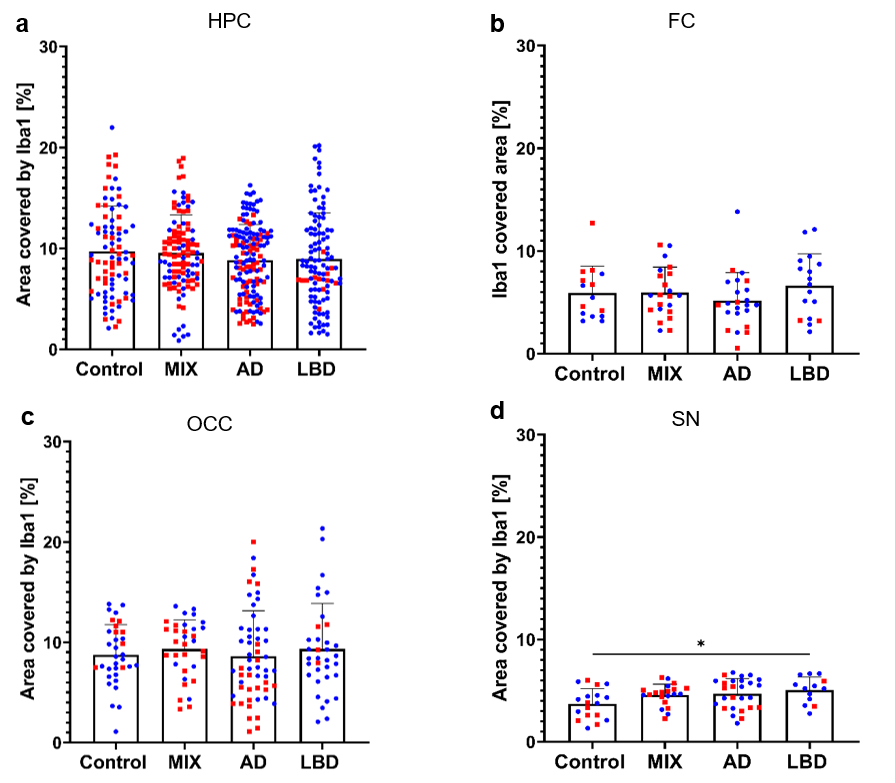


**Supplementary figure S3.** **Quantification of area covered by Iba1 pos. cell. a** – **d** Iba1 pos. stained microglia quantified in Control, AD, MIX and LBD cases for: **a** – HPC (hippocampus), **b** – FC (frontal cortex), **c** – OCC (occipital cortex) and **d** – SN (substantia nigra). **Red** – female, **blue** – male. No sex differences could be shown across the examined brain regions. One-way ANOVA with Tukey's multiple comparisons test was used for statistical analysis. *p < 0.05, **p < 0.01, ***p < 0.001.


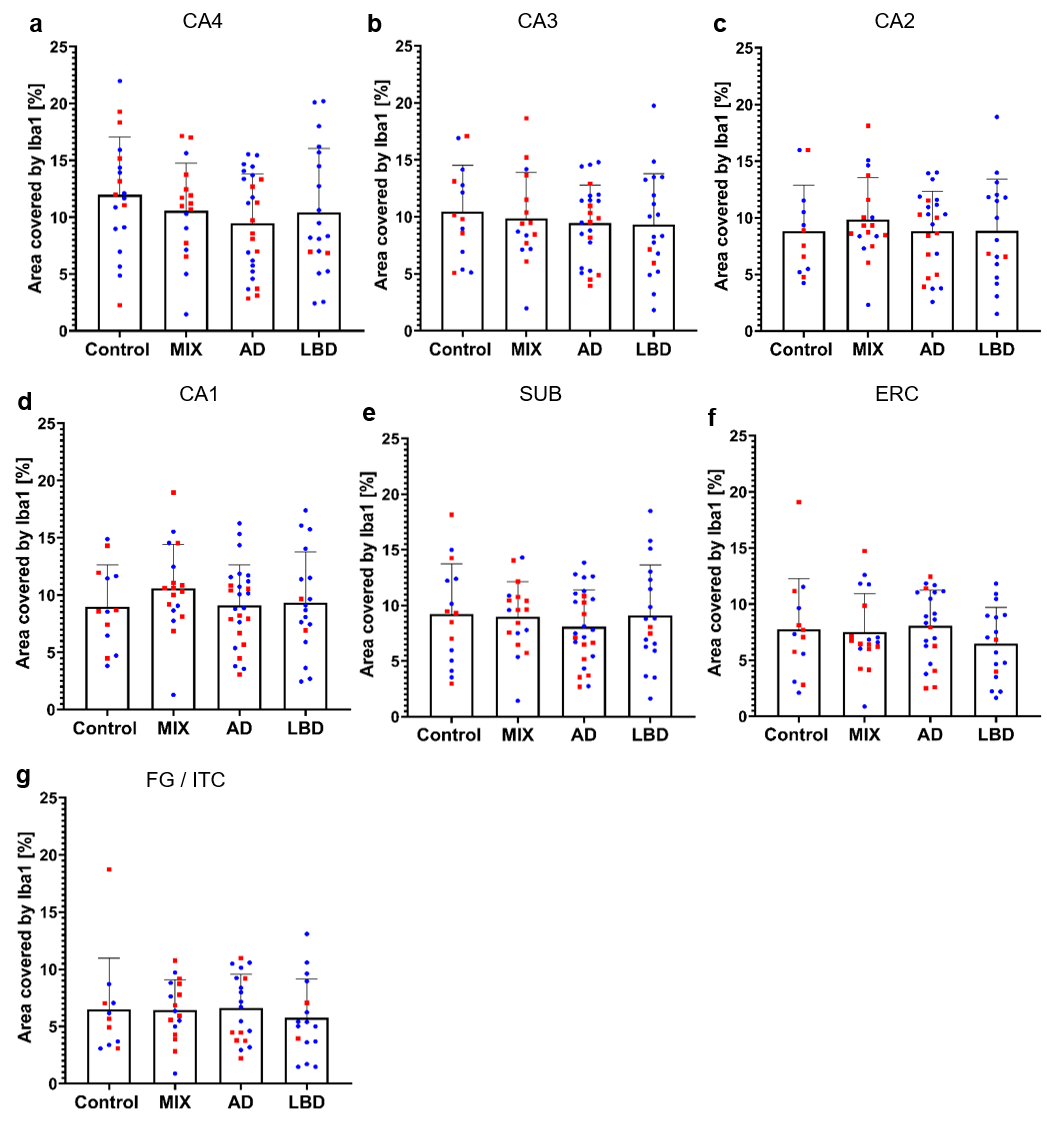


**Supplementary figure S4.** **Quantification of area covered by Iba1 pos. cell in hippocampal subregions. a** – **g** Iba1 pos. stained microglia in CA (cornu ammonis) 4 -1, SUB (subiculum), ERC (entorhinal cortex), FG/ITC (fusiform gyrus / inferior temporal cortex). **Red** – female, **blue** – male. No sex differences could be shown across the examined brain regions. One-way ANOVA with Tukey's multiple comparisons test was used for statistical analysis. *p < 0.05, **p < 0.01, ***p < 0.001.


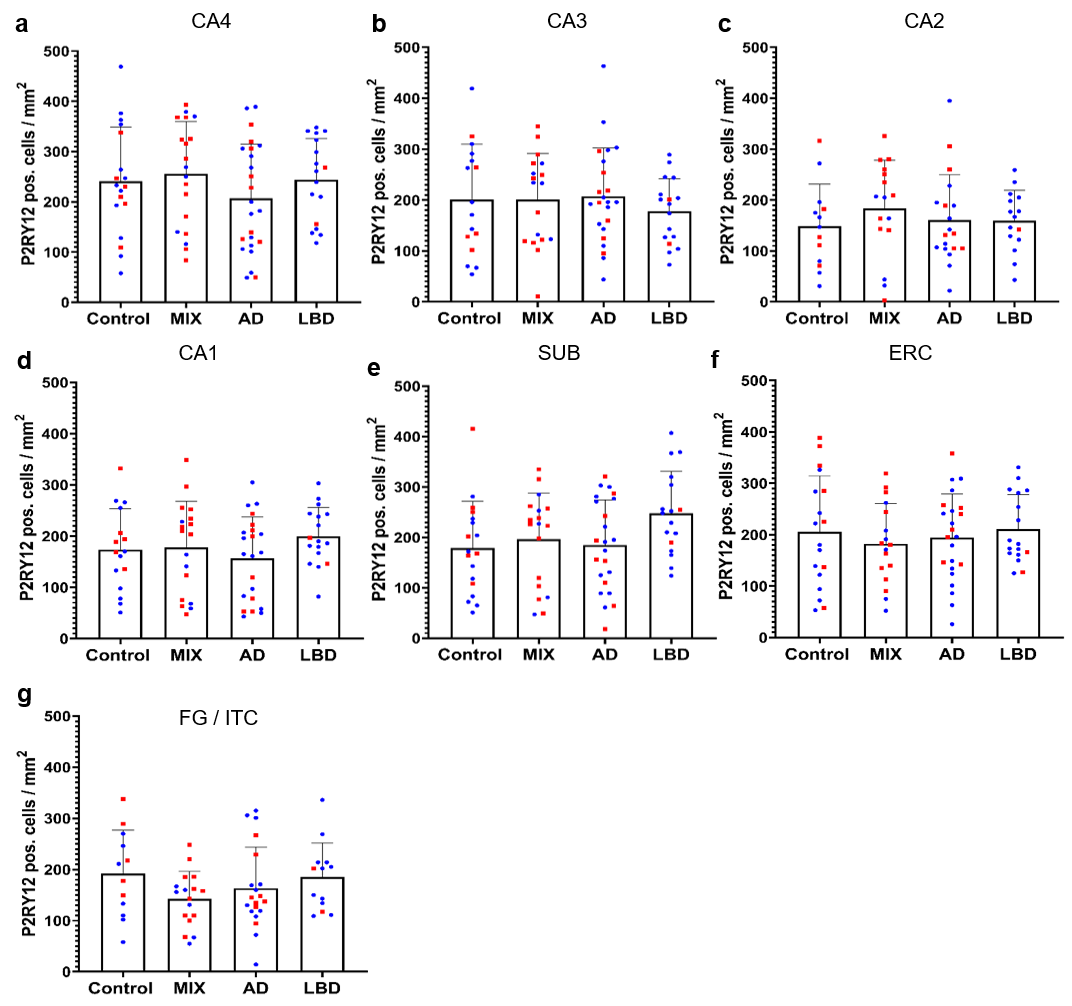


**Supplementary figure S5.** **Quantification of P2RY12 pos. cell in hippocampal subregions. a** – **g** Counted P2RY12 pos. cells in CA (cornu ammonis) 4 -1, SUB (subiculum), ERC (entorhinal cortex), FG/ITC (fusiform gyrus / inferior temporal cortex). **Red** – female, **blue** – male. No sex differences could be shown across the examined brain regions. One-way ANOVA with Tukey's multiple comparisons test was used for statistical analysis. *p < 0.05, **p < 0.01, ***p < 0.001.


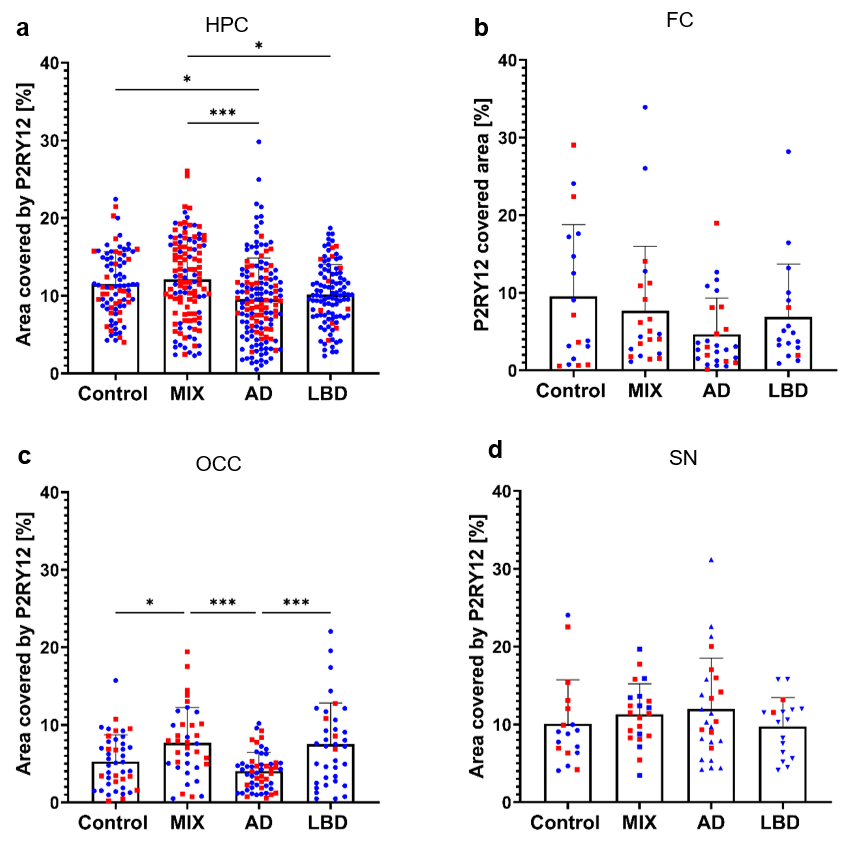


**Supplementary figure S6.** **Quantification of area covered by P2RY12 pos. cell. a** – **d** P2RY12 pos. stained microglia quantified in Control, AD, MIX and LBD cases for: **a** – HPC (hippocampus), **b** – FC (frontal cortex), **c** – OCC (occipital cortex) and **d** – SN (substantia nigra). **Red** – female, **blue** – male. No sex differences could be shown across the examined brain regions. One-way ANOVA with Tukey's multiple comparisons test was used for statistical analysis. *p < 0.05, **p < 0.01, ***p < 0.001.


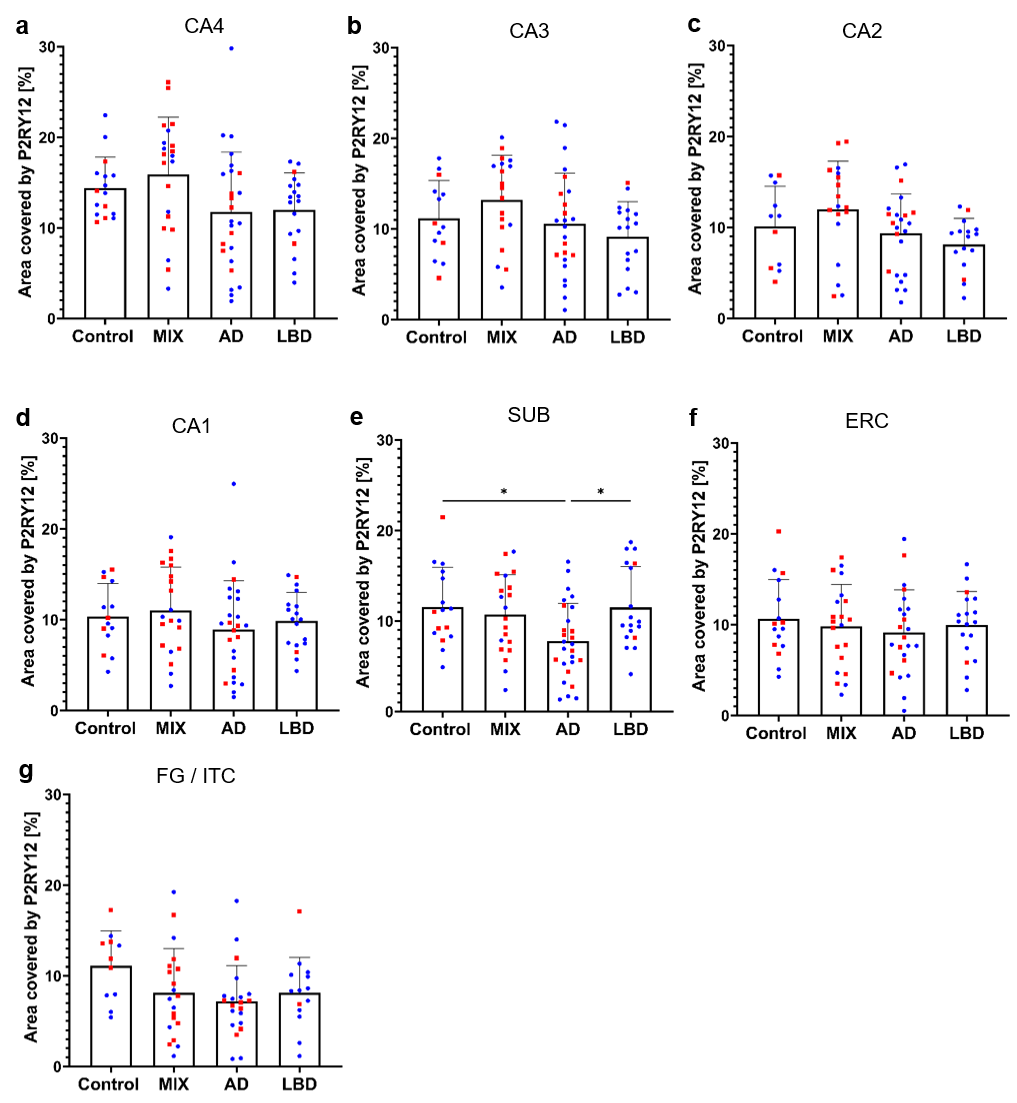


**Supplementary figure S7.** **Quantification of area covered by P2RY12 pos. cell in hippocampal subregions. a** – **g** P2RY12 pos. stained microglia in CA (cornu ammonis) 4 -1, SUB (subiculum), ERC (entorhinal cortex), FG/ITC (fusiform gyrus / inferior temporal cortex). **Red** – female, **blue** – male. No sex differences could be shown across the examined brain regions. One-way ANOVA with Tukey's multiple comparisons test was used for statistical analysis. *p < 0.05, **p < 0.01, ***p < 0.001.


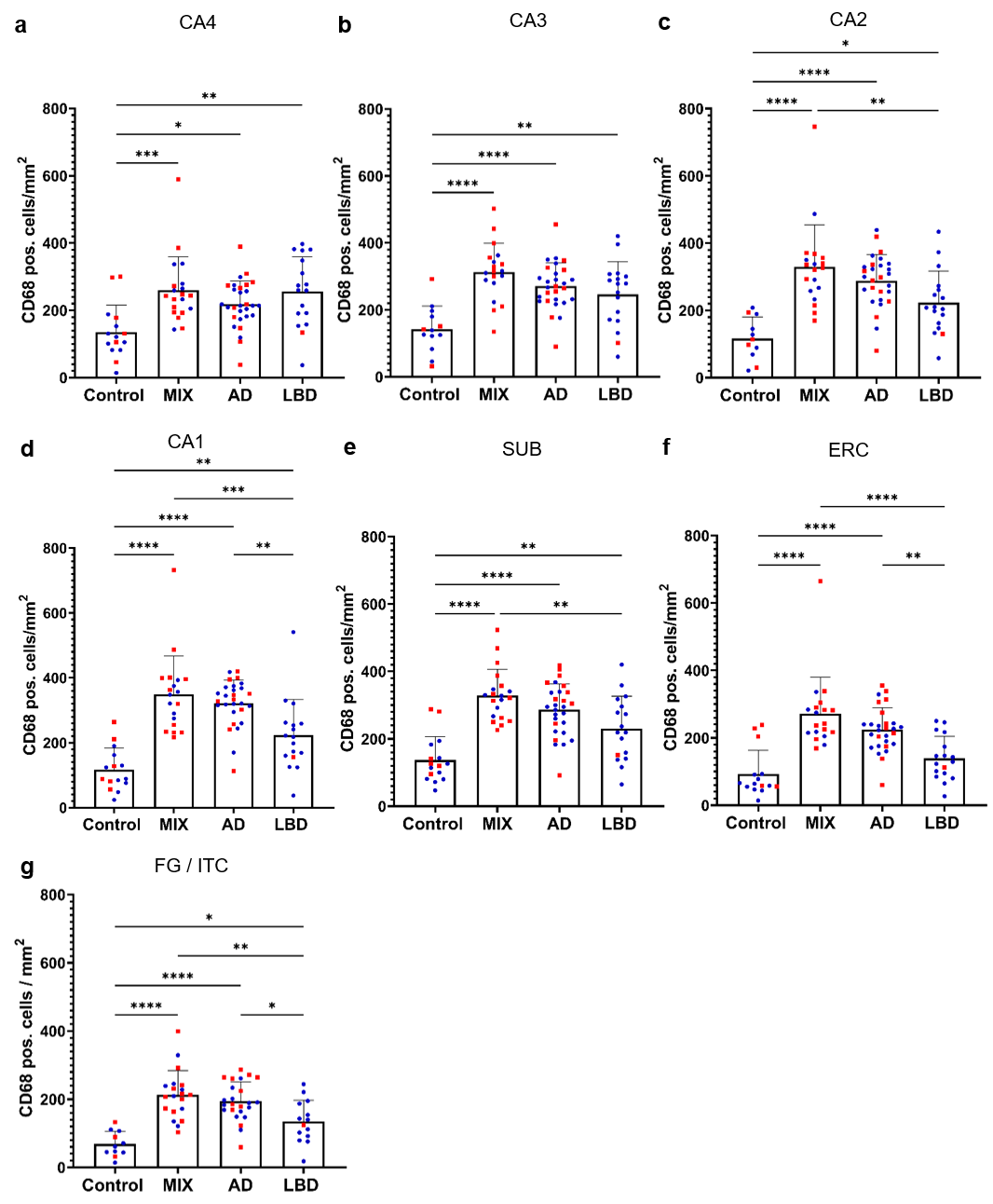


**Supplementary figure S8.** **Quantification of CD68 pos. cell in hippocampal subregions. a** – **g** Counted CD68 pos. cells in CA (cornu ammonis) 4 -1, SUB (subiculum), ERC (entorhinal cortex), FG/ITC (fusiform gyrus / inferior temporal cortex). **Red** – female, **blue** – male. No sex differences could be shown across the examined brain regions. One-way ANOVA with Tukey's multiple comparisons test was used for statistical analysis. *p < 0.05, **p < 0.01, ***p < 0.001.


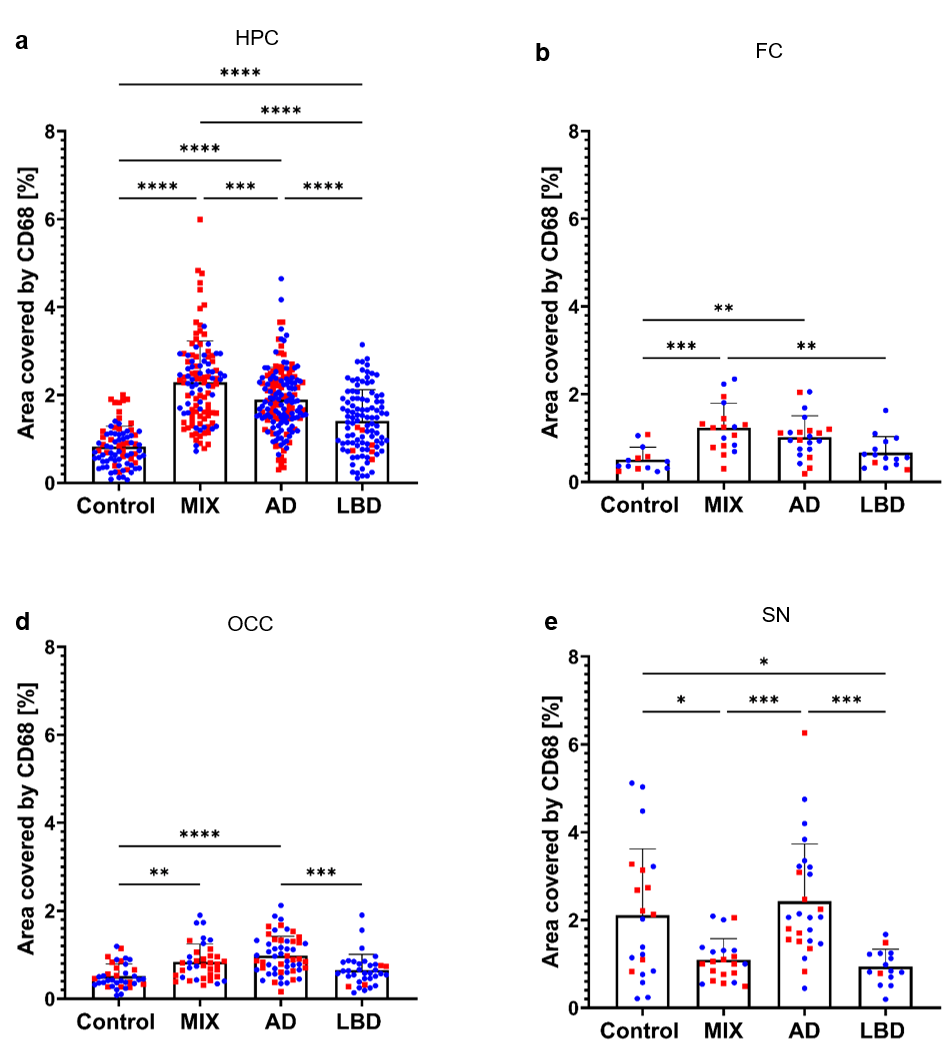


**Supplementary figure S9.** **Quantification of area covered by CD68 pos. cell. a** – **d** CD68 pos. stained microglia quantified in Control, AD, MIX and LBD cases for: **a** – HPC (hippocampus), **b** – FC (frontal cortex), **c** – OCC (occipital cortex) and **d** – SN (substantia nigra). **Red** – female, **blue** – male. No sex differences could be shown across the examined brain regions. One-way ANOVA with Tukey's multiple comparisons test was used for statistical analysis. *p < 0.05, **p < 0.01, ***p < 0.001.


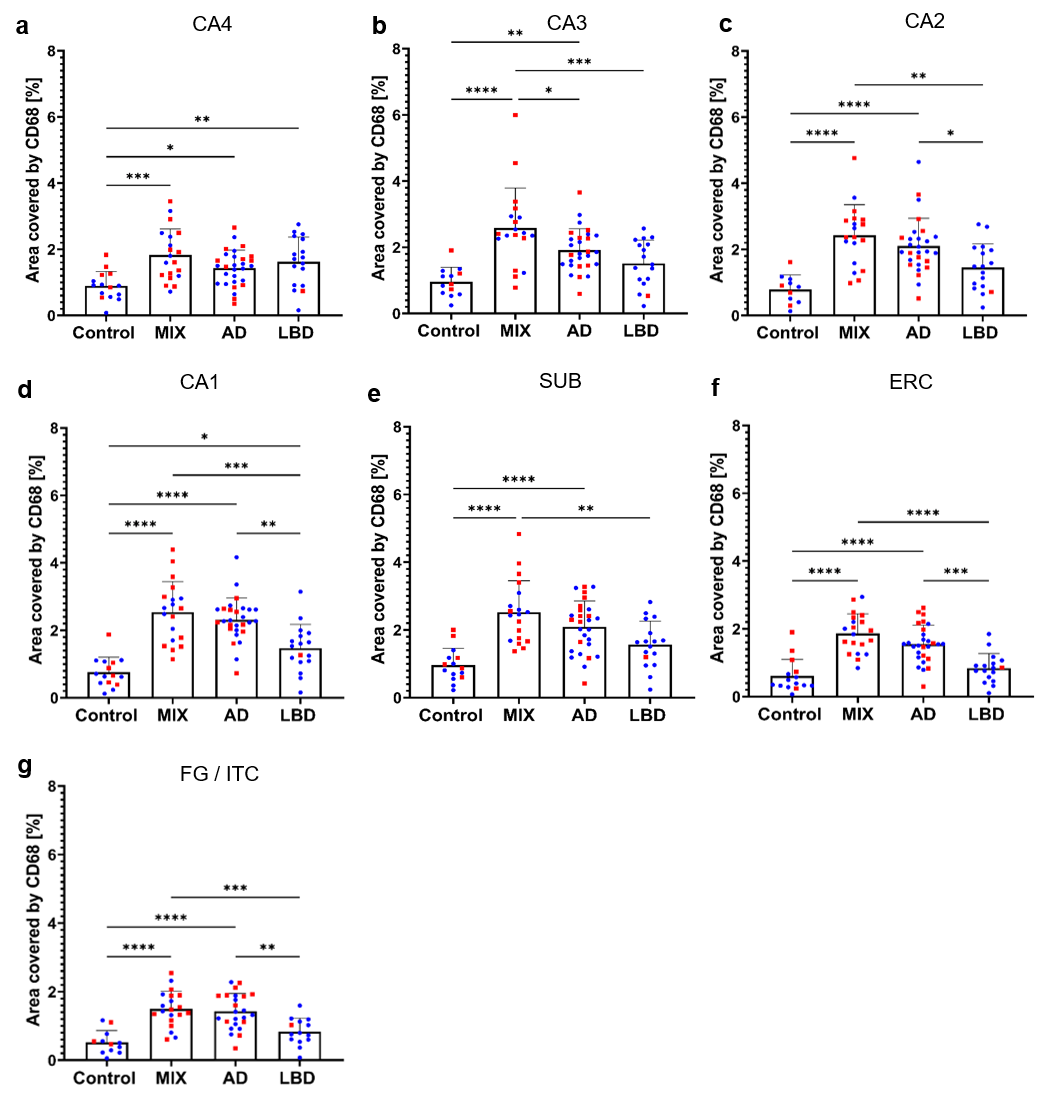


**Supplementary figure S10.** **Quantification of area covered by CD68 pos. cell in hippocampal subregions. a** – **g** CD68 pos. stained microglia in CA (cornu ammonis) 4 -1, SUB (subiculum), ERC (entorhinal cortex), FG/ITC (fusiform gyrus / inferior temporal cortex). **Red** – female, **blue** – male. No sex differences could be shown across the examined brain regions. One-way ANOVA with Tukey's multiple comparisons test was used for statistical analysis. *p < 0.05, **p < 0.01, ***p < 0.001.


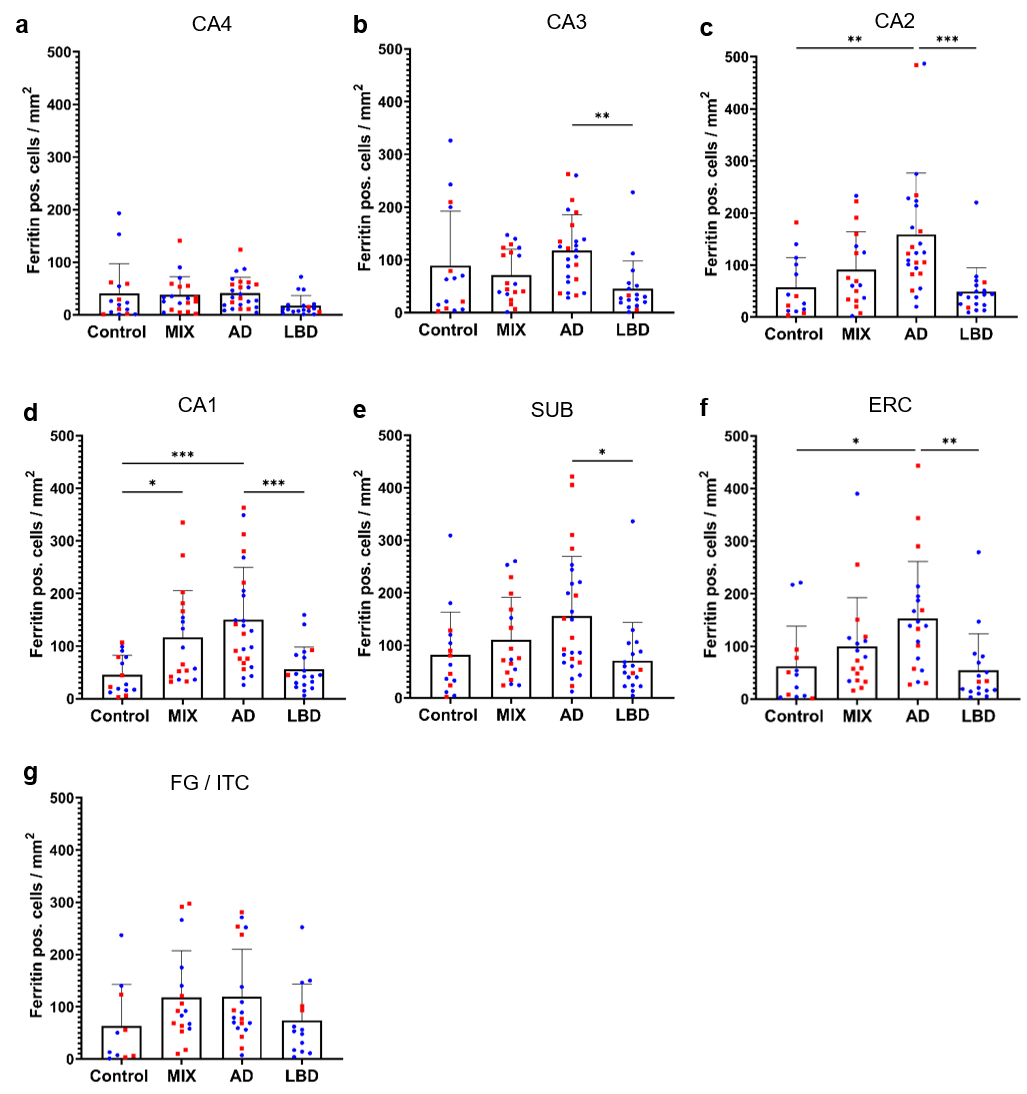


**Supplementary figure S11.** **Quantification of Ferritin pos. cell in hippocampal subregions. a** – **g** Counted Ferritin pos. cells in CA (cornu ammonis) 4 -1, SUB (subiculum), ERC (entorhinal cortex), FG/ITC (fusiform gyrus / inferior temporal cortex). **Red** – female, **blue** – male. No sex differences could be shown across the examined brain regions. One-way ANOVA with Tukey's multiple comparisons test was used for statistical analysis. *p < 0.05, **p < 0.01, ***p < 0.001.

**
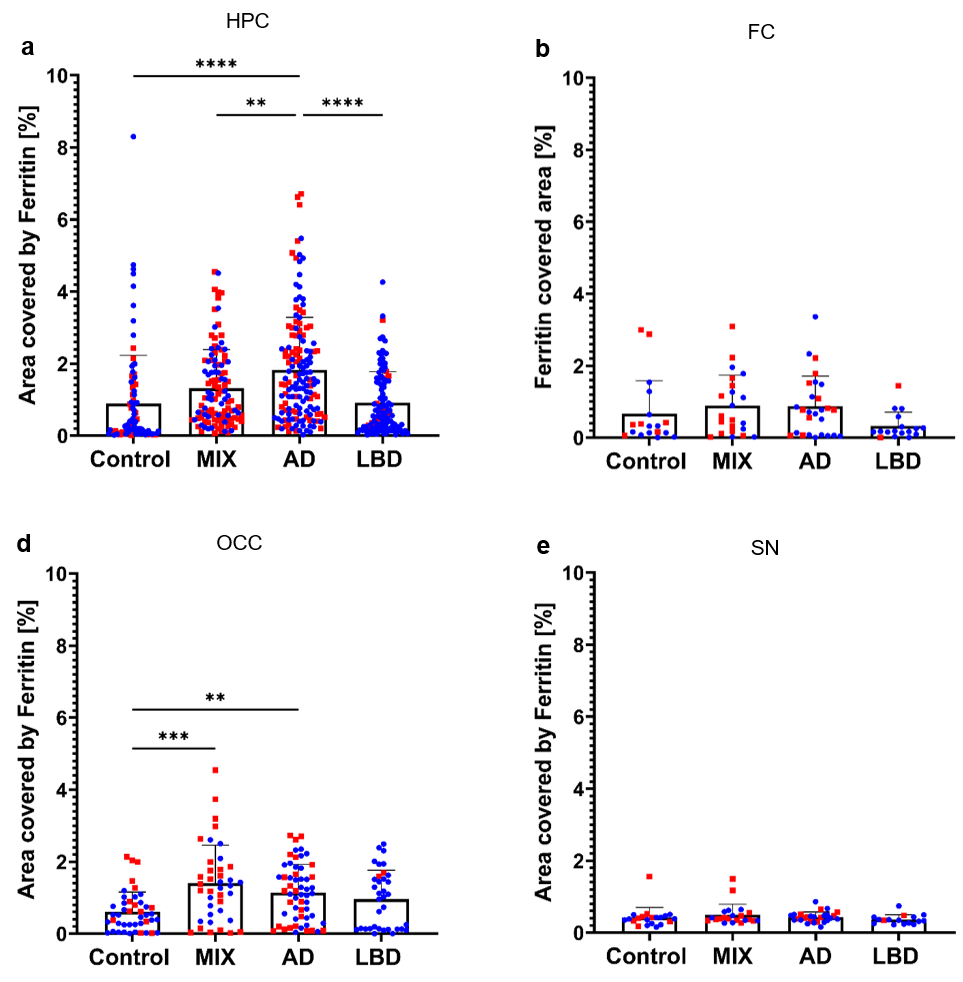
**

**Supplementary figure S12.** **Quantification of area covered by Ferritin pos. cell. a** – **d** Ferritin pos. stained microglia quantified in Control, AD, MIX and LBD cases for: **a** – HPC (hippocampus), **b** – FC (frontal cortex), **c** – OCC (occipital cortex) and **d** – SN (substantia nigra). **Red** – female, **blue** – male. No sex differences could be shown across the examined brain regions. One-way ANOVA with Tukey's multiple comparisons test was used for statistical analysis. *p < 0.05, **p < 0.01, ***p < 0.001.


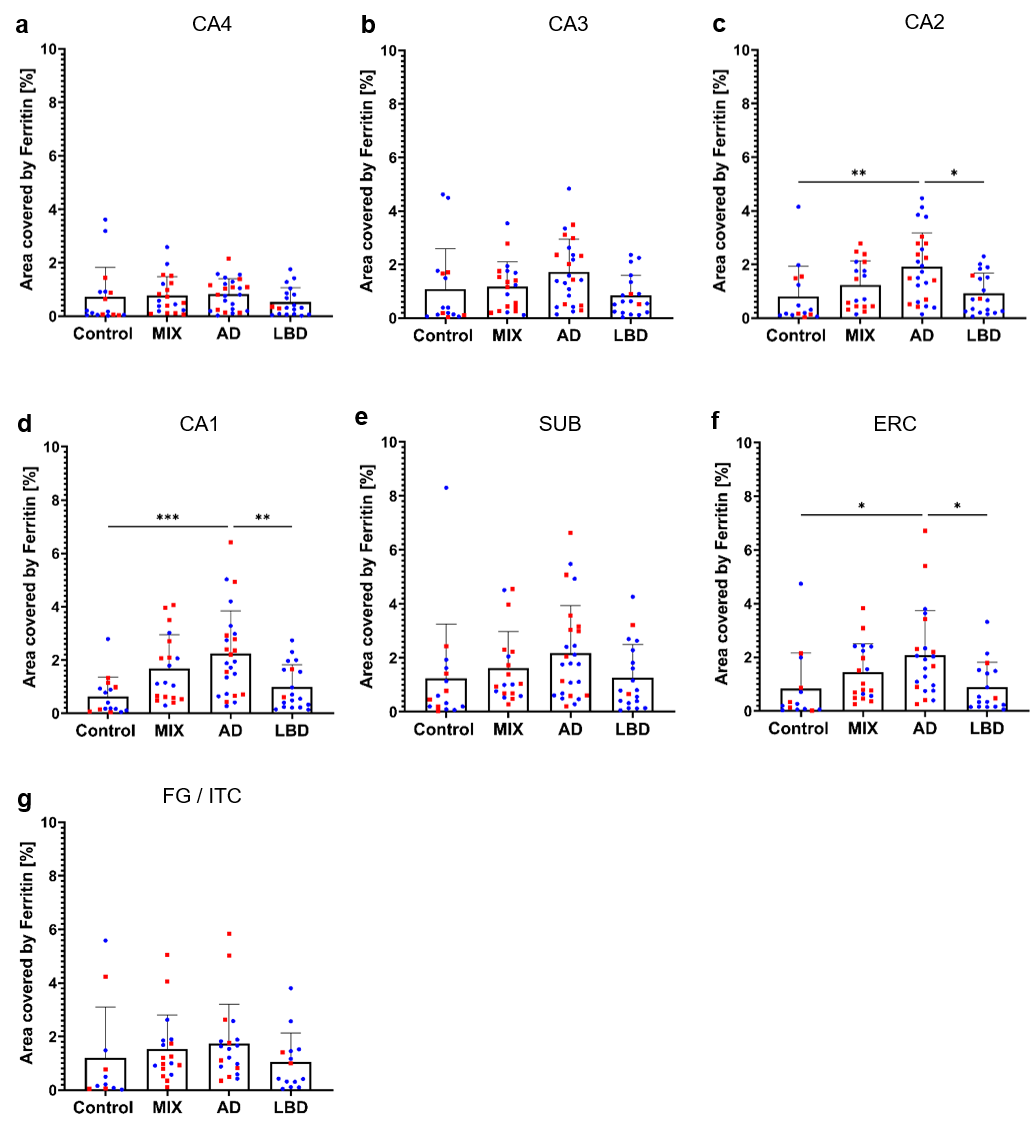


**Supplementary figure S13.** **Quantification of area covered by Ferritin pos. cell in hippocampal subregions. a** – **g** Ferritin pos. stained microglia in CA (cornu ammonis) 4 -1, SUB (subiculum), ERC (entorhinal cortex), FG/ITC (fusiform gyrus / inferior temporal cortex). **Red** – female, **blue** – male. No sex differences could be shown across the examined brain regions. One-way ANOVA with Tukey's multiple comparisons test was used for statistical analysis. *p < 0.05, **p < 0.01, ***p < 0.001.


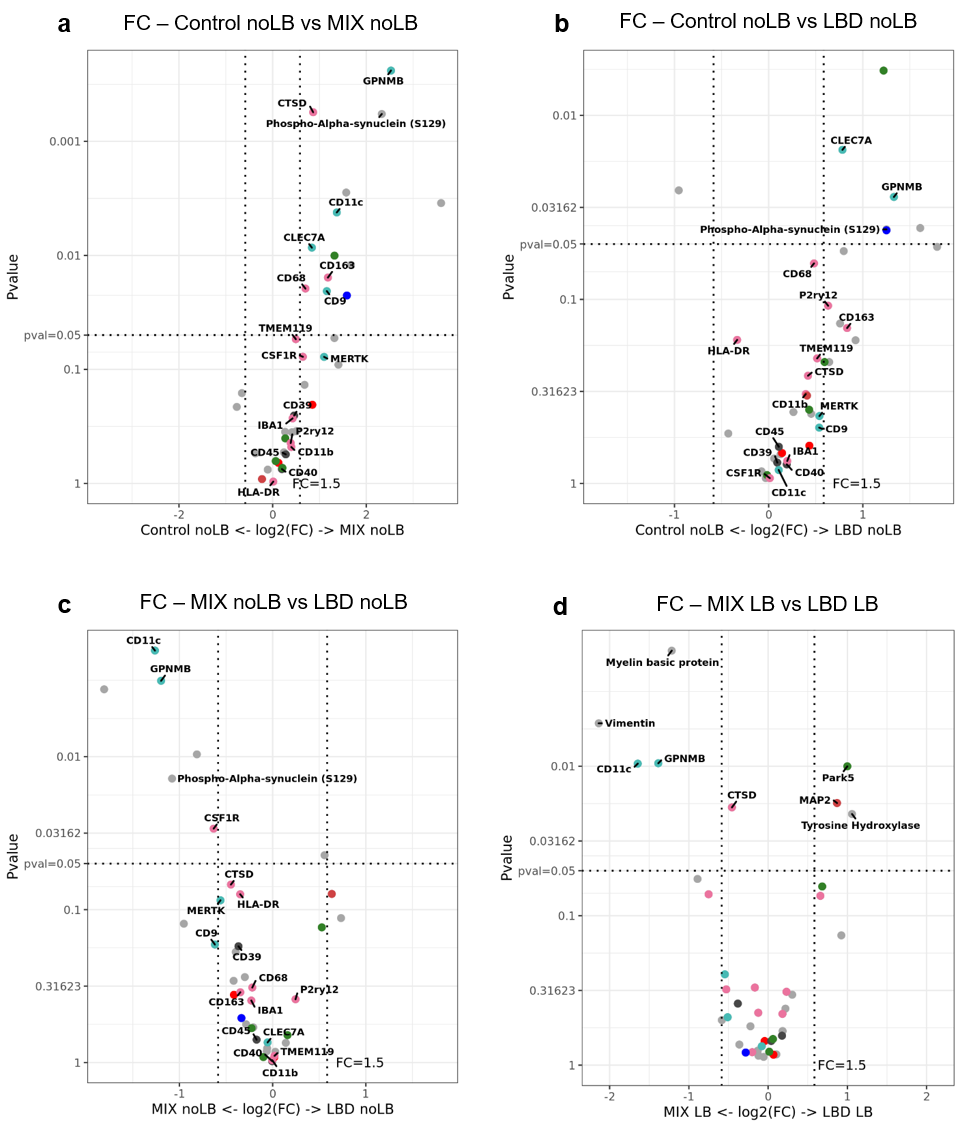


**Supplementary figure S14.** **Digital spatial profiling (DSP) of the microenvironment of Lewy body bearing and non-Lewy body bearing neurons in frontal cortex.** **a** – **d** Volcano plot for the expressed proteins. Comparison of the microenvironment of Lewy body bearing neurons (MIX and LBD) and neurons without pathology (Control): **a** – microenvironment of non-Lewy body bearing neurons in Control and MIX, **b** – Control and LBD, **c** – MIX and LBD. **d** - Volcano plot for the expressed proteins in the microenvironment of Lewy body bearing neurons in MIX and LBD. In volcano plots, unadjusted p‐value of 0.05 and fold‐change (FC) of 1.5 were used to identify differentially expressed proteins. Color code of detected proteins based on used NanoString Antibody Panels: blue – AD, red – Astrocyte/Inflammation, green – PD, pink – Microglia, turquoise – Disease-Associated Microglia, dark grey – Microglia/Inflammation, light grey – other.


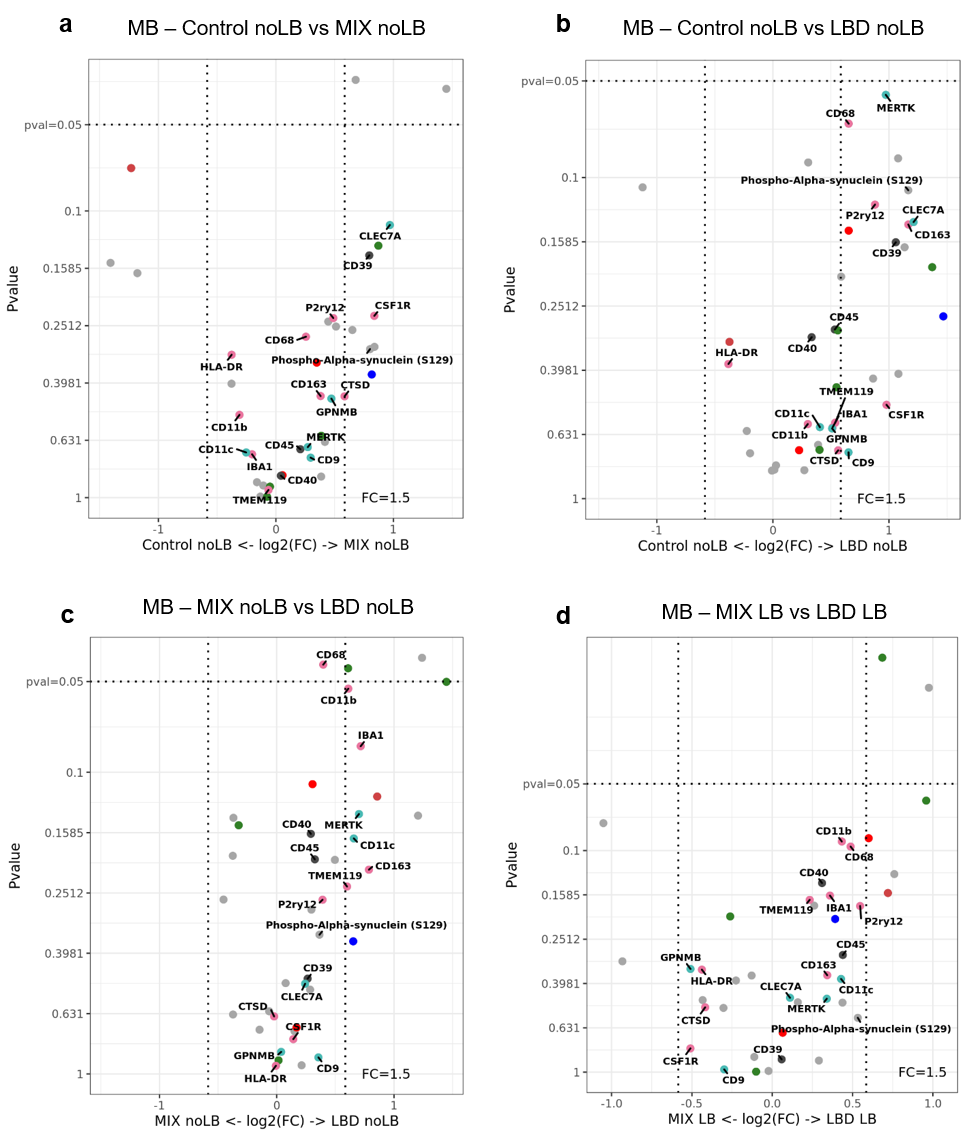


**Supplementary figure S15.** **Digital spatial profiling (DSP) of the microenvironment of Lewy body bearing and non-Lewy body bearing neurons in midbrain.** **a** – **d** Volcano plot for the expressed proteins. Comparison of the microenvironment of Lewy body bearing neurons (MIX and LBD) and neurons without pathology (Control): **a** – microenvironment of non-Lewy body bearing neurons in Control and MIX, **b** – Control and LBD, **c** – MIX and LBD. **d** - Volcano plot for the expressed proteins in the microenvironment of Lewy body bearing neurons in MIX and LBD. In volcano plots, unadjusted p‐value of 0.05 and fold‐change (FC) of 1.5 were used to identify differentially expressed proteins. Color code of detected proteins based on used NanoString Antibody Panels: blue – AD, red – Astrocyte/Inflammation, green – PD, pink – Microglia, turquoise – Disease-Associated Microglia, dark grey – Microglia/Inflammation, light grey – other.


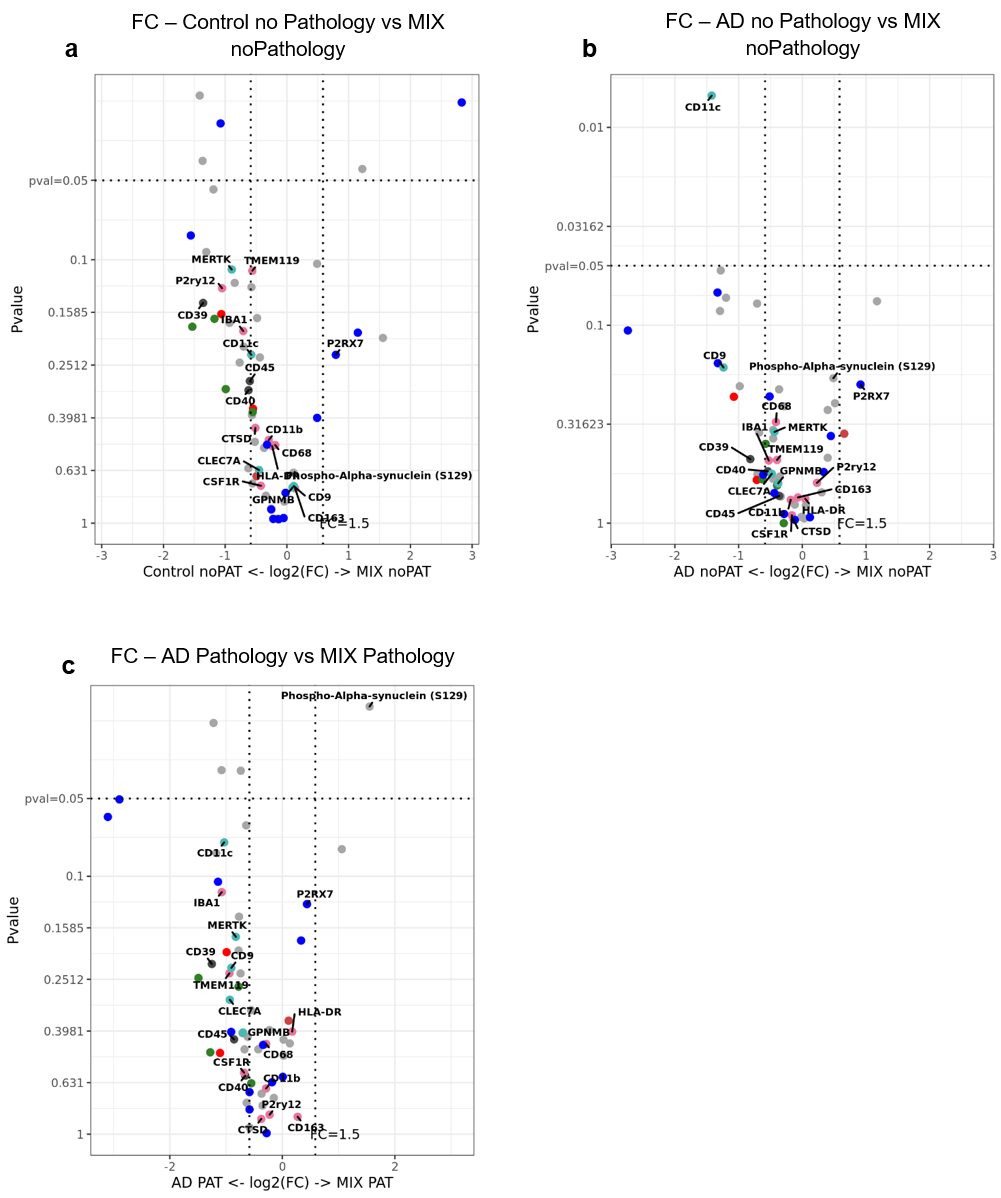


**Supplementary figure S16.** **Digital spatial profiling (DSP) of the microenvironment of neurons with and without pathology.** **a** – **c** Volcano plot for the expressed proteins. Comparison of the microenvironment of neurons with pathology (AD and MIX) and neurons without pathology (Control): **a** – microenvironment of pathology-free neurons in Control and MIX, **b** – AD and MIX. **c** – Volcano plot for the expressed proteins in the microenvironment of neurons with ADNC pathology in AD and MIX. In volcano plots, unadjusted p‐value of 0.05 and fold‐change (FC) of 1.5 were used to identify differentially expressed proteins. Color code of detected proteins based on used NanoString Antibody Panels: blue – AD, red – Astrocyte/Inflammation, green – PD, pink – Microglia, turquoise – Disease-Associated Microglia, dark grey – Microglia/Inflammation, light grey – other.


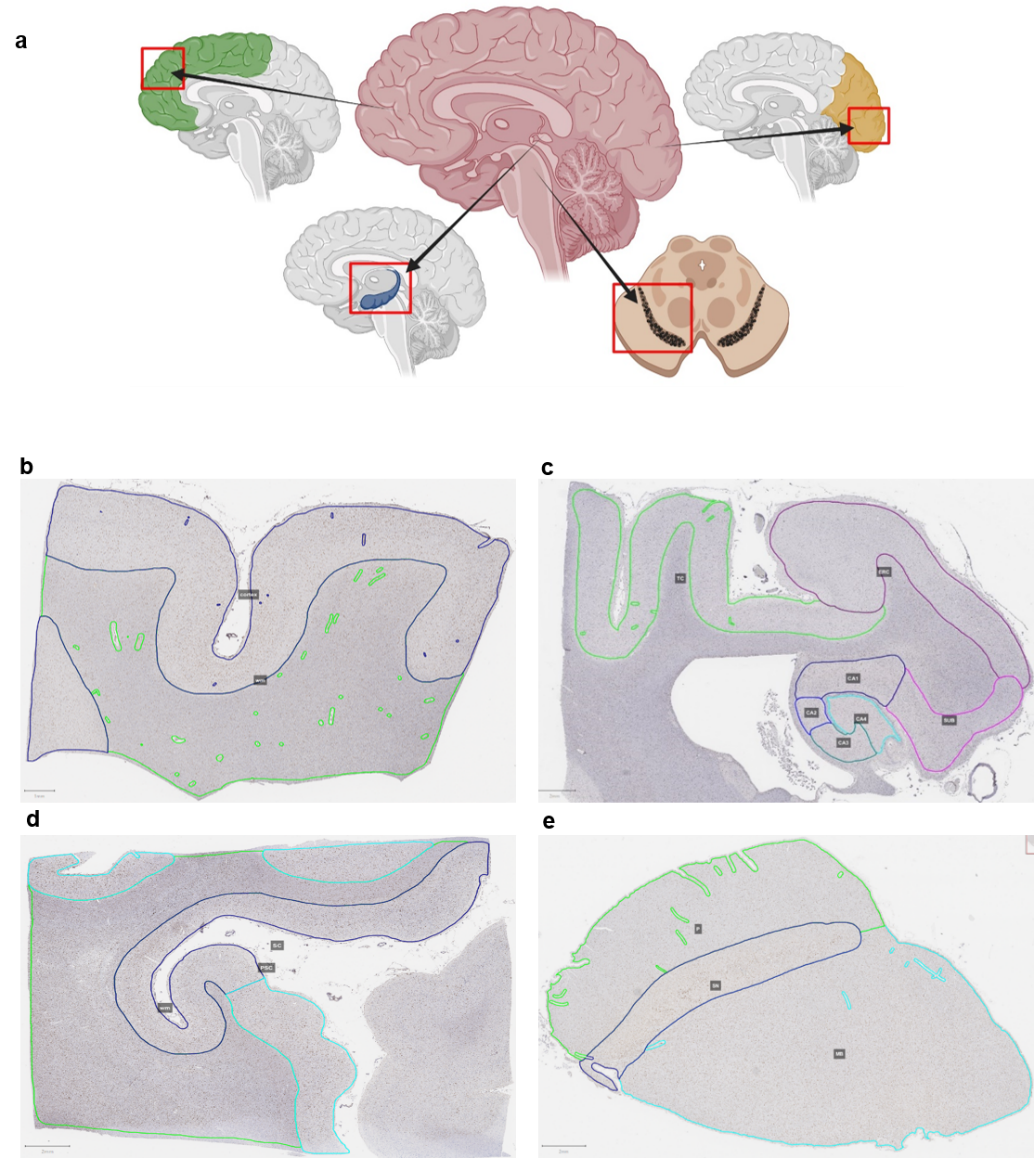


**Supplementary figure S17.** **Overview of selected regions and areas for experiments and quantification.** **a** – Diagram of the brain regions examined (HPC, FC, OCC, MB). **b** – **e** Areas selected for quantification with QuPath. The areas for annotation were selected for the gray matter in frontal cortex (**b**) and occipital cortex (**d**). Hippocampus (**c**) was divided into subregions: CA4 - CA1, subiculum (SUB) and entorhinal cortex (ERC). For midbrain (**e**), the areas were defined as substantia nigra (SN), peduncle (P) and other midbrain area (MB). Figure created with BioRender.


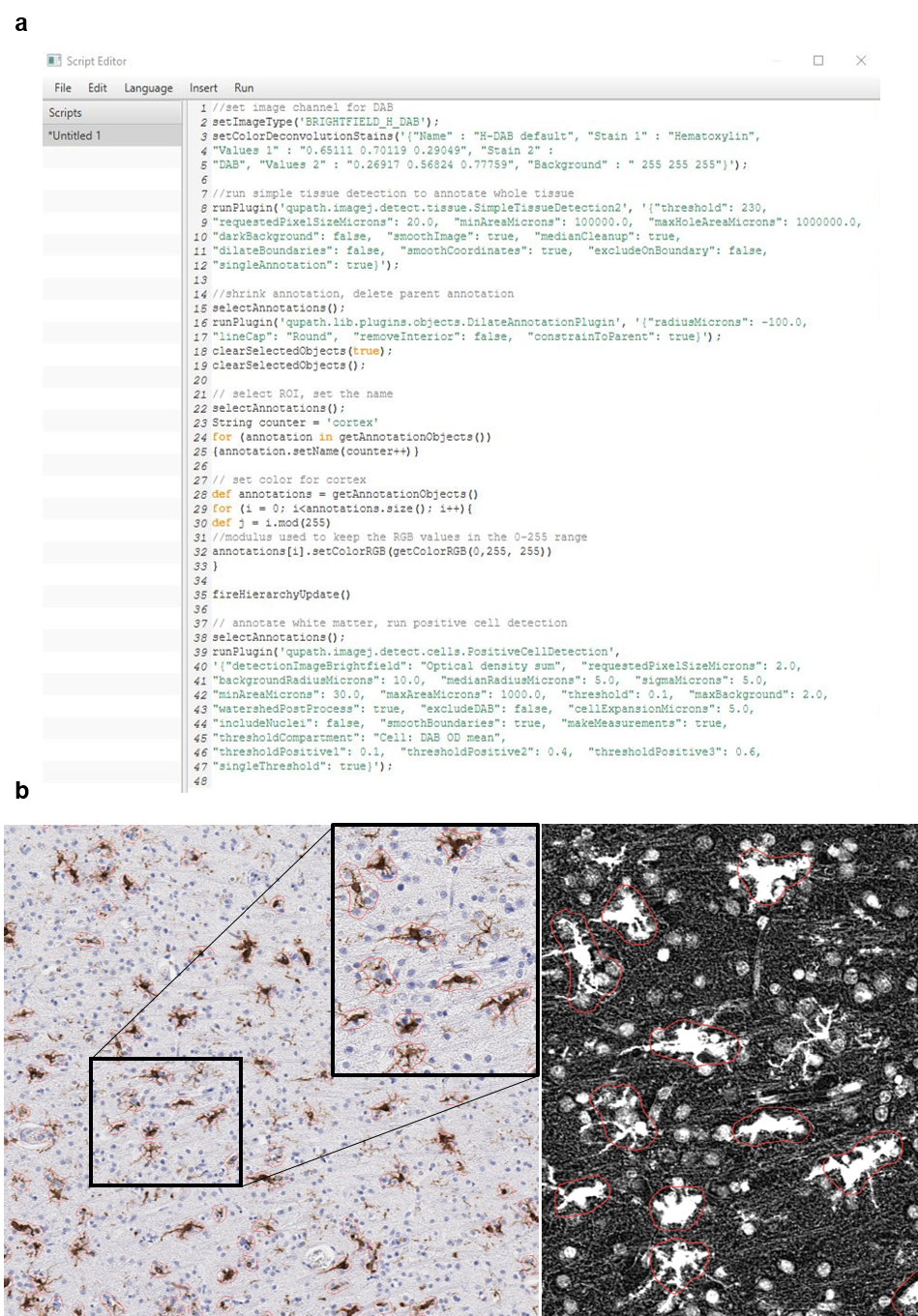


**Supplementary figure S18.** **Script and overview of the positive cell detection tool for the quantification of microglia.** **a** – Script used in QuPath to detect DAB stained microglia in human brain tissue. Values specified in the script for detection. **b** – Detection of cells using the positive cell detection tool. Thresholds were determined by user inspection of detected cells. Red circles indicate cells that were detected as positive.


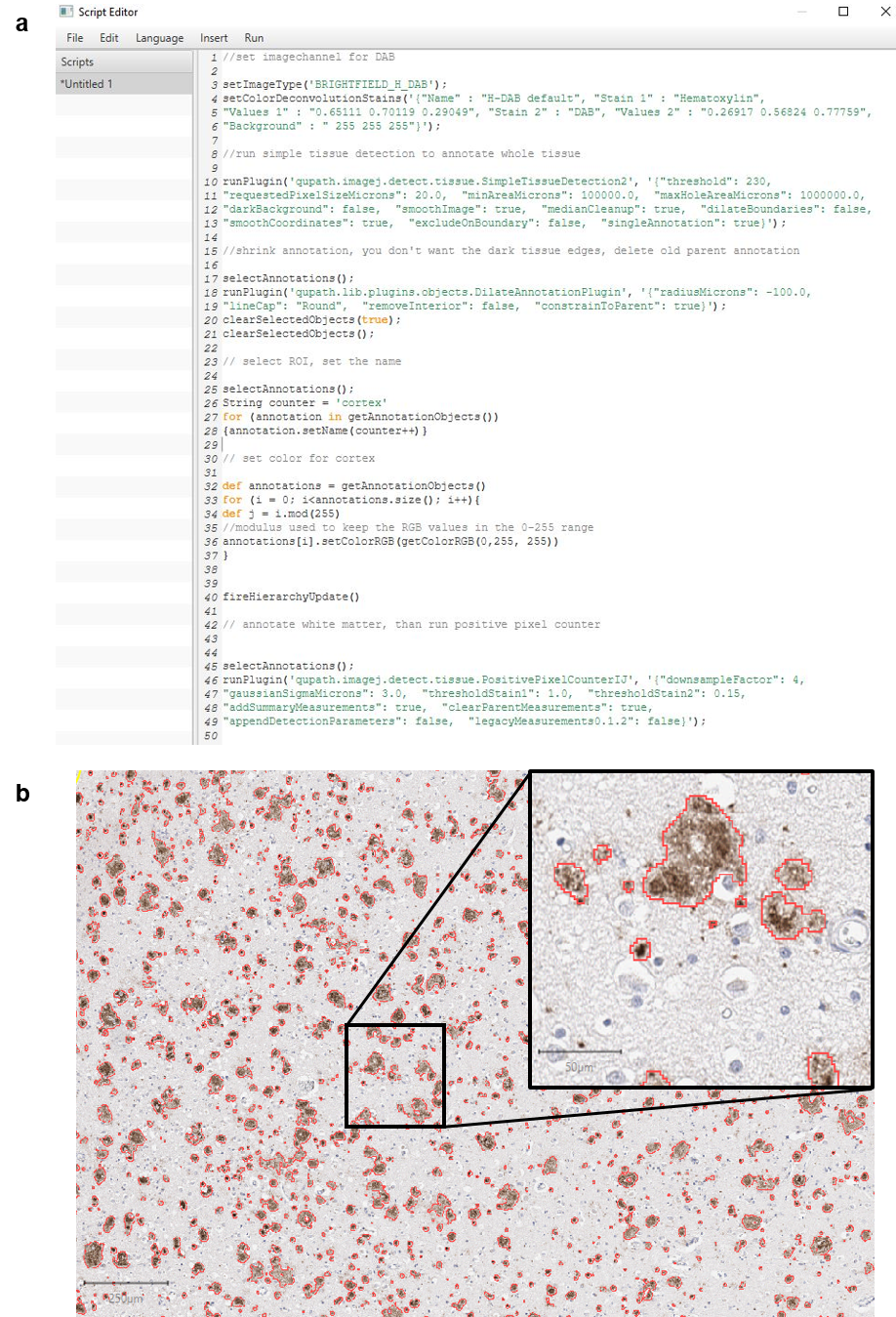


**Supplementary figure S19.** **Script and overview of the positive pixel detection tool for the quantification of protein pathologies.** **a** – Script used in QuPath to detect DAB stained pathologies in human brain tissue. Values specified in the script for detection. **b** – The positive pixel detection tool calculates the area covered by pixels above a certain threshold value (result: area covered in %). Red circles indicate detected pixels in an accumulation of protein pathology. Amyloid beta representative for analyzed pathologies.


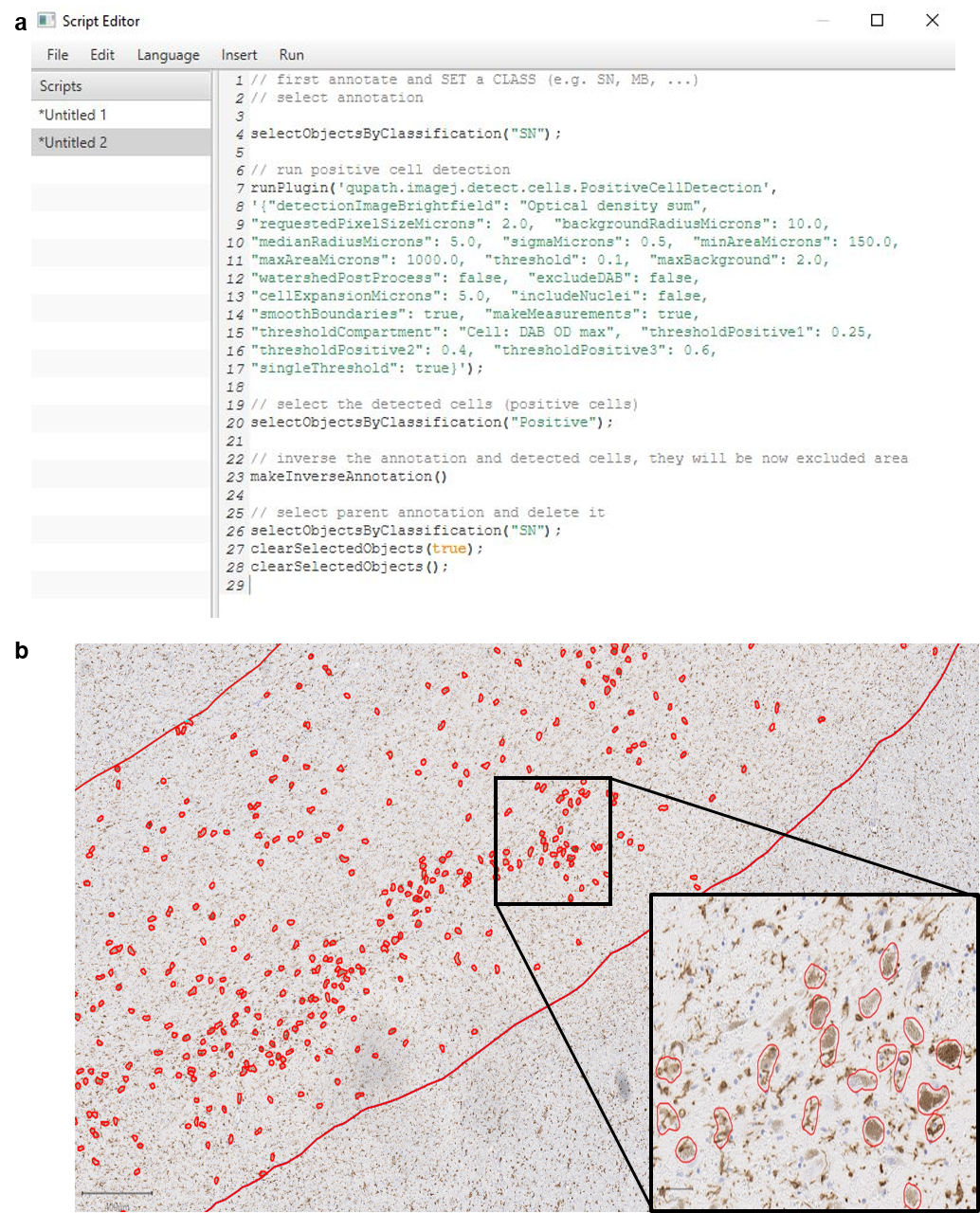


**Supplementary figure S20** **Overview of the used script to exclude dopaminergic neurons in the substantia nigra tissue.** **a** – Script used in QuPath to detect dopaminergic neurons and exclude them from the annotation. Due to their natural coloration, these neurons would be counted positively and influence the result of the quantification. Values specified in the script for detection., **b** – Detected dopaminergic neurons in the substantia nigra are recognized and excluded from the annotated area. Red circles indicate detected neurons that are excluded.


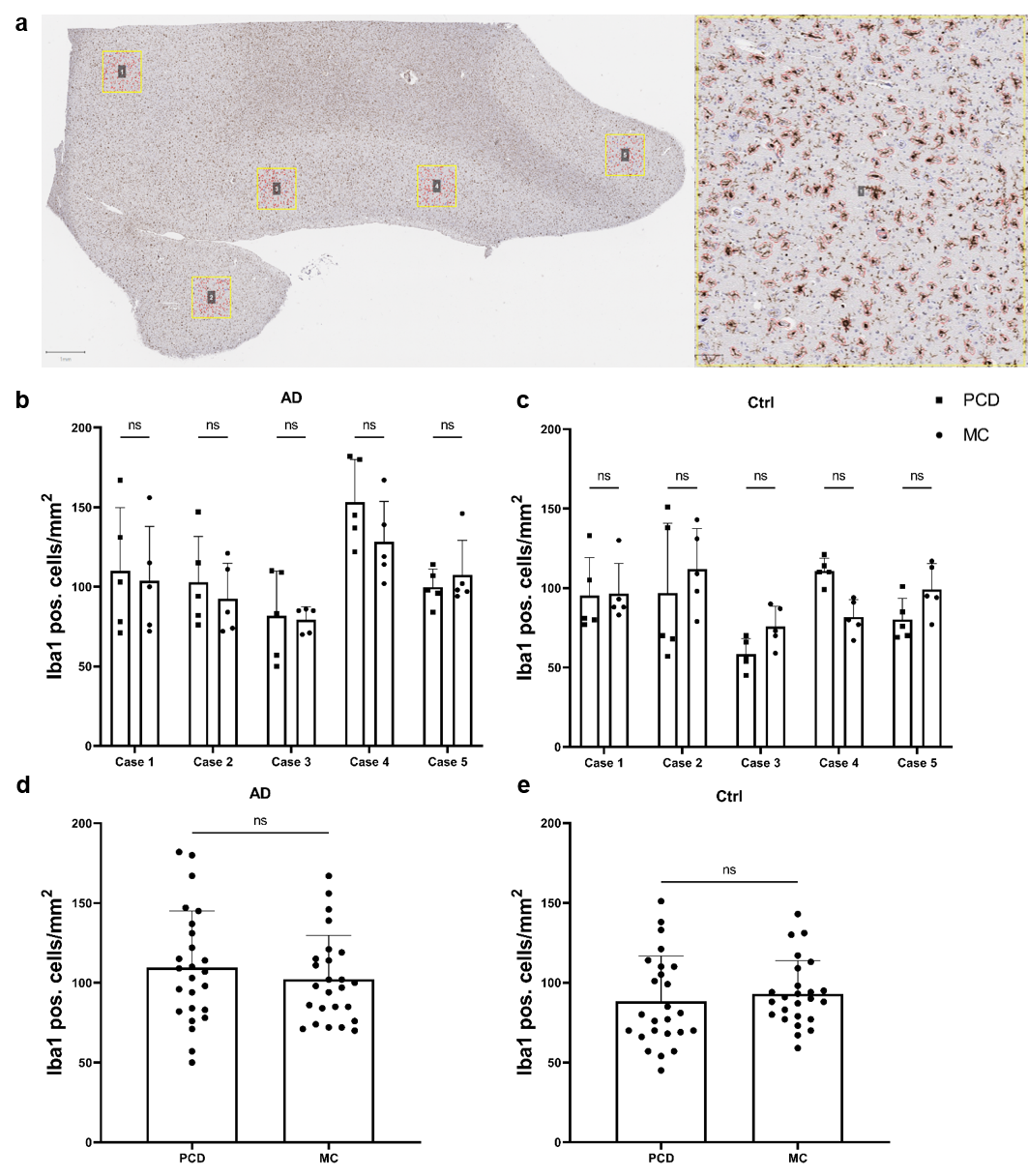


**Supplementary figure S21** **Comparison between manual cell count (MC) and positive cell detection tool (PCD).** **a** – Overview of annotation selection. Five identical squares were randomly placed in the gray matter area of five AD and Control cases. Squares were 1 mm x 1 mm in size. Microglia in the squares were counted manually and with QuPath’s PCD. **b – e** Comparison of manually counted microglia with the number counted with PCD. Dots in the bars of the graph represent the annotation boxes for each case (**b – c**). Two-way ANOVA with Sidak's multiple comparison test was used for statistical analysis. **d – e** Paired t-test for manual and software-assisted cell counting in AD (**d**) and Control (**e**) cases.
